# MetaMine – A tool to detect and analyse gene patterns in their environmental context

**DOI:** 10.1186/1471-2105-9-459

**Published:** 2008-10-28

**Authors:** Uta Bohnebeck, Thierry Lombardot, Renzo Kottmann, Frank O Glöckner

**Affiliations:** 1 ttz Bremerhaven, An der Karlstadt 10, D-27568 Bremerhaven, Germany; 2 Max Planck Institute for Marine Microbiology, Celsiusstraße 1, D-28359 Bremen, Germany; 3 Jacobs University Bremen, Campus Ring 1, 28759 Bremen, Germany

## Abstract

**Background:**

Modern sequencing technologies allow rapid sequencing and bioinformatic analysis of genomes and metagenomes. With every new sequencing project a vast number of new proteins become available with many genes remaining functionally unclassified based on evidences from sequence similarities alone. Extending similarity searches with gene pattern approaches, defined as genes sharing a distinct genomic neighbourhood, have shown to significantly improve the number of functional assignments. Further functional evidences can be gained by correlating these gene patterns with prevailing environmental parameters. MetaMine was developed to approach the large pool of unclassified proteins by searching for recurrent gene patterns across habitats based on key genes.

**Results:**

MetaMine is an interactive data mining tool which enables the detection of gene patterns in an environmental context. The gene pattern search starts with a user defined environmentally interesting key gene. With this gene a BLAST search is carried out against the Microbial Ecological Genomics DataBase (MEGDB) containing marine genomic and metagenomic sequences. This is followed by the determination of all neighbouring genes within a given distance and a search for functionally equivalent genes. In the final step a set of common genes present in a defined number of distinct genomes is determined. The gene patterns found are associated with their individual pattern instances describing gene order and directions. They are presented together with information about the sample and the habitat. MetaMine is implemented in Java and provided as a client/server application with a user-friendly graphical user interface. The system was evaluated with environmentally relevant genes related to the methane-cycle and carbon monoxide oxidation.

**Conclusion:**

MetaMine offers a targeted, semi-automatic search for gene patterns based on expert input. The graphical user interface of MetaMine provides a user-friendly overview of the computed gene patterns for further inspection in an ecological context. Prevailing biological processes associated with a key gene can be used to infer new annotations and shape hypotheses to guide further analyses. The use-cases demonstrate that meaningful gene patterns can be quickly detected using MetaMine.

MetaMine is freely available for academic use from .

## Background

More than 99% of the microbial diversity on earth still resists cultivation. To address their metabolic potential, numerous efforts to clone and sequence large DNA-fragments directly from the environment (the metagenome) have been accomplished worldwide. Several studies [1-3] have shown that on average only for 27–48% of the genes a specific function can be inferred by similarity searches [4]. This untapped pool of so called hypothetical proteins represents a still unexploited source for new enzymatically driven reactions.

With the availability of a large number of complete genomes comparative genomics becomes increasingly important. The major aspect of this approach is the analysis of gene neighbourhoods to indicate functional association, which can therefore significantly improve the predictions of gene functions. This idea was first systematically applied in 1999 by Overbeek and colleagues [5]. They introduced the concept of a "pair of close bidirectional best hits" and could prove functional coupling of such genes for several pathways. Some years later the subsystem approach was introduced [6,7], which is a generalization of the pathway concept and describes a group of related functional roles jointly involved in a specific aspect of the cellular machinery. Today systems like IMG [8]/IMG/M [9], STRING [10,11] and RAST [12] provide several functionalities to analyse gene neighbourhoods and other protein interaction features for a broad range of microbial genomes based on pre-computed data.

Additional support for functional evidences might be gained by correlating these gene patterns with prevailing environmental parameters. The growing number of genome and metagenome sequences from the environment, especially from the marine system, opens for the first time the possibility to link genomic information with environmental parameters in a systematic way [13-15]. Subsequently, if process correlated gene patterns can be identified in the habitats it should be possible to return hints on the functions of the respective patterns.

MetaMine is an interactive data mining tool which enables the detection of gene patterns, defined as genes sharing a distinct genomic neighbourhood, initiated by a user provided key gene. The underlying pipeline was designed to handle genomic data sets where gene family classification information is not available or incomplete. Because consistent family classification is a prerequisite for the pattern determination step we first calculate gene groups of functional equivalence. This is still an open research problem. Therefore, our system allows the user to work with different parameter settings and to switch between alternative methods. By focussing on the resulting patterns the calculation of the functional groups allows the inclusion of highly similar paralogs and genes to be part in several groups, because errors made in this step can be easily revealed by the gene patterns found. Given a user selected key gene of environmental relevance MetaMine carries out a semi-automatic search for gene patterns on a regularly updated database of marine genomes and metagenomes. The gene patterns found are presented to the user together with information about the samples and the habitat within an interactive graphical user interface (GUI) for further inspection. To our knowledge, currently no system exists combining genomic and metagenomic pattern information with environmental parameters.

## Implementation

### Microbial Ecological Genomics Database

The Microbial Ecological Genomics DataBase (MEGDB) contains prokaryotic genome and metagenome sequences of marine origin together with information about their environmental context. A list of criteria have been used to select the (meta-)genomic sequences to build the database: i) public access (sequences must be available in one of the public sequence databases [16]); ii) marine origin; iii) bacterial or archaeal; iv) high sequence quality (i.e. assembled contigs with a sequencing coverage of at least eight fold) and v) the exact geographic origin of the sequences (e.g. from the original publication).

Habitat parameters like water and sediment depths, temperature, salinity, and other physical-chemical properties have been extracted from the literature or extrapolated based on global ocean data sets like the World Ocean Atlas and the World Ocean Database [17] and remote sensing information (SeaWiFS) [18] within the EU project MetaFunctions [19]. A detailed overview of the current content of MEGDB can be found at .

Besides MetaMine, public access to the MEGDB is granted by the MetaLook tool [20] and especially the Genomes Mapserver as a central entry point [14,21]. A Geographic-BLAST tool is also available online to get an overview of the geographical distribution of particular genes across the world.

### Gene patterns and key gene approach

The term "gene pattern" often covers two related biological observations in genetics/genomics. In prokaryotic genomes, genes are often organised in operons, where transcription leads to a single messenger RNA molecule (mRNA) encoding different subunits of a protein or even distinct, but related proteins. The definition of an operon, as a set of commonly regulated genes is strict, but as long as no common mRNA is proven experimentally, the corresponding set of genes is often called a gene cluster/gene pattern which is loosely defined as a set of neighbouring genes with possibly coupled functions and/or conserved order across organisms.

The distinction between operons and gene patterns is not crucial for the biological questions we want to address with MetaMine. Moreover, the detection of habitat specific gene patterns requires some extensions with respect to environmental parameters in the concept described above. For the systematic search for correlations between the habitat and the gene content two basic types of gene patterns are of interest: (1) genes which are present or over/under-represented under specific environmental conditions and (2) patterns consisting of a set of genes occurring in specific genomic neighbourhoods. If such gene patterns are found genomic context analysis assists in potential functional assignments. In addition, if gene patterns correlate with distinct environmental parameters or processes further evidences for potential functions may be inferred. MetaMine was designed to detect such gene patterns. Due to the huge amount of genomic and metagenomic sequences we decided to apply a bottom-up approach, where prior biological knowledge is used to select a so-called *key gene *with known biological function and environmental relevance which plays the role of a seed to search for gene patterns in genomic sequences with at least two predicted genes.

### Process steps

For gene pattern discovery the user can carry out the following process steps starting with the selection of the key gene. A detailed description of the analysis process including a flowchart can be found in the user guide available on the website and as Additional file 2.

#### 1. & 2. Definition of a project and a key gene

In order to store and retrieve the results of a certain analysis the corresponding processing steps are organised in a project described by a project name, the user and a short comment. Also the key gene is defined by a name, a short description of its function and a comment.

#### 3. Import/retrieval of the corresponding key gene sequence

The corresponding key gene sequence(s) can be imported from an external file containing the protein sequence in Fasta-format or by retrieving the protein sequence from the MEGDB.

#### 4. BLAST search with key gene

Using the key gene sequence as a query for a BLAST search [22] against all marine genomic and metagenomic sequences with at least two predicted genes stored in the MEGDB is carried out. The result is a table with information about *e*-value, organism, sampling site, habitat, water and sediment depths and potential gene functions of similar genes found by this BLAST search presented to the user in a specific BLAST panel within the MetaMine GUI.

#### 5. Determination of neighbouring genes

Given a user-defined parameter *k *the *k *neighbouring genes to each side with respect to all the genes found by the BLAST search in the previous step are determined and shown in tabular form. Using mouseover as well as a second panel the user can see detailed information about the functional annotation of the genes.

#### 6. BLAST search of all neighbouring genes

A BLAST search is carried out with all neighbouring genes from the last step. The results are represented by a hash table containing the set of all neighbouring genes together with their associated BLAST results. The user can access this hash table by clicking a gene in the neighbour table and gets the associated BLAST information in the corresponding BLAST panel.

#### 7. Determination of functionally equivalent genes

**Figure 1:**
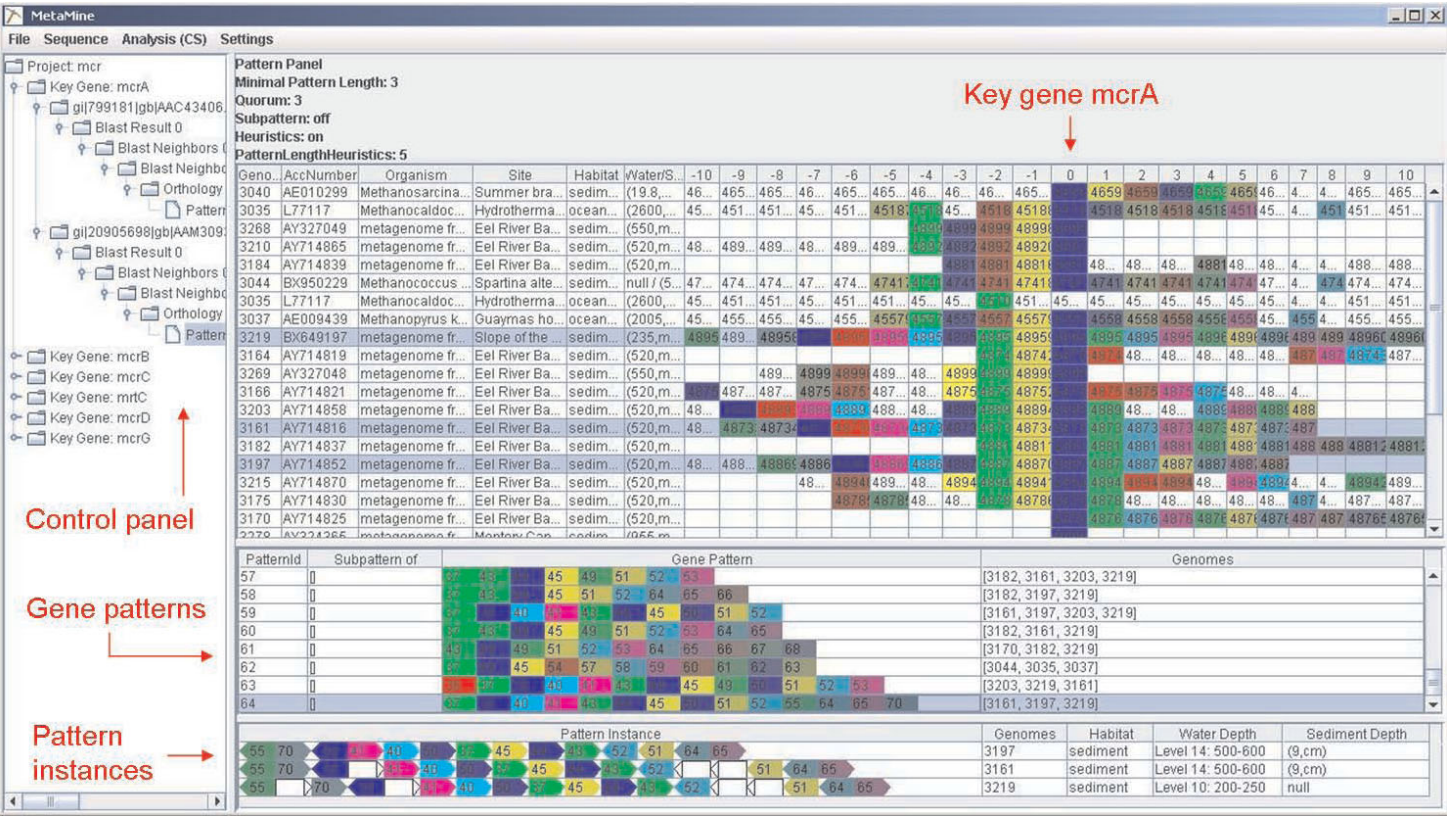
MetaMine screenshot of the pattern panel.

In order to detect functionally equivalent genes a reciprocal best hit approach followed by a clustering algorithm is carried out. The result is a set of groups, whereby each group represents genes of functional equivalence. All group members are colour-coded and presented to the user in the table of neighbouring genes (Fig. 1). In addition, the reciprocal best matches and the functional groups are shown in separate views.

#### 8. Determination of gene patterns

Given two user-defined parameters minimal length *l *and quorum *q *a gene pattern is defined as a set of at least *l *genes (functional groups) which are all present in at least *q *different genomes or metagenome samples. Each pattern is associated with a pattern instance view. Pattern instances describing also gene order and directions are shown with their environmental information in tabular form (see also Fig 1). Guidelines for parameter settings can be found in the user guide (see Additional file 2).

#### 9. Storage and retrieval of all intermediate results

All intermediate results are organised in special data objects which can be stored to and retrieved from the local MEGDB (stand-alone version only) as well as exported to and imported from external XML files.

Each process step can be repeated with other parameters resulting in a tree structure to organise intermediate results. As shown in the left panel of Fig. 1, the user can navigate through the history of all steps to analyse the corresponding results in detail. The user should be aware that the final results can be influenced by the methods and parameter settings of all previous steps. Therefore, the differences can be used to prove the stability of the results. In case a certain gene is not in the functional group or gene pattern as expected this roll back mechanism allows further in depth analysis.

If the user specifies all parameters in advance he can also start a batch-mode analysis. All parameters can be adjusted using the Parameter Dialog "Set Parameters" in the Settings menu.

### Algorithms

The following section gives a short overview of the underlying algorithms describing basic ideas and strategies.

#### Determination of functionally equivalent genes

In this step we are interested in finding groups of functionally equivalent genes which constitute the elements for the next step – the determination of common gene patterns. Different concepts and methods to obtain such groups exist. The classical and well established method – introduced by Clusters of Orthologous Groups (COG) [23] – relies on the phylogeny-based concept of orthology. Orthology describes genes in different species that have derived from a common ancestor by speciation in contrast to paralogous genes which arise from a duplication event. Therefore, orthology represents a strong relationship with a high potential describing the same biological function. Nevertheless, it is a phylogenetic concept originally introduced to study gene evolution. Consequently this excludes paralogs which might still have same functions. A complementary concept is to model intrinsic properties for gene function derived from multiple alignments and domain architecture as it is applied by Pfam [24,25]. A third variant are automatic methods based on sequence similarity and unsupervised clustering like TRIBES [26].

A large proportion of our data set consists of metagenome samples with a high potential of new gene sequences not present in existing databases which might form new and sometimes small functional groups. Therefore, we started with the basic idea of COG and relaxed the constraints for metagenomes and inparalogs [27]. In general orthology is a well established concept for functional annotation and with the establishment of the gene patters the potential error of including some false positives is easily ruled out. In this context Boekhorst and Snel [28] have shown that "sharing gene order and similarity in size dramatically increases the chance of a query-hit pair being homologous".

To detect the groups of functionally equivalent genes we use a heuristic approach restricted to the gene sequences found in the BLAST searches which consist mainly of the following two steps:

• determination of reciprocal best matches and

• determination of groups of functionally equivalent genes.

Let *G *denote a set of identifiers for all genome and metagenome sequences which are stored in MEGDB and associated with an organism name and a sampling site, respectively and *R *denote a set of identifiers for all sequence regions which are predicted to be a protein encoding gene, then *genome*: *R *→ *G *is a function determining the genome identifier for a certain gene. Let *b*_*r *_denote the result of a BLAST search for gene *r *∈ *R *against the MEGDB and *B *a set of BLAST results *b*_*r *_for a given set *R*, then *rbm*: *R *× *B *× *G *→ *R *is a function determining the identifier of the reciprocal best BLAST hit for gene *r *∈ *R *with respect to genome *g *∈ *G*.

A reciprocal best match is commonly defined as follows: Gene *g*_*a *_in genome *G*_*A *_is the best match of gene *g*_*b *_in genome *G*_*B *_and gene *g*_*b *_is the best match of gene *g*_*a*_. Given the set of BLAST results *B *the function *rbm *checks this constraint for the genes and genomes specified. The search for reciprocal best matches is restricted to the set *R*_*n *_⊂ *R *representing the genes found in the BLAST search with the key gene and their neighbours. Therefore, *R*_*n *_corresponds to the upper table in the right panel in Fig. 1. In addition, the set of genomes *G*_*n *_⊂ *G *is restricted to ∪_*r *∈ *Rn *_*genome*(*r*). These are the genomes related to the genes (and their neighbours) found in the BLAST search with the key gene, which correspond to the rows of this table. The result of this step is a hash map *RBM *with key *r *∈ *R*_*n *_storing a vector of genome related reciprocal best matches *rbm *= (*rbm*_*g*1_, *rbm*_*g*2_, ..., *rbm*_*gn*_) with ∀*g*_*i *_∈ *G*_*n *_for each gene from the neighbour table. This intermediate result can be seen in the second table in the Orthology Panel.

The next step is the determination of functional groups which is based only on the information stored in the hash map *RBM *and carried out in a bottom-up manner. Let *F *denote this set of functional groups where each group *f*_*i *_∈ *F*, *i *= 1, ..., |*F*| contains a set of functionally equivalent genes, they are established as follows:

• For each gene *r *∈ *R*_*n *_the corresponding vector of genome related reciprocal best matches is retrieved from the hash map *RBM *and checked for triangle relationships of reciprocal best matches. If such a triangle relationship exists between at least three genes of the vector a potential group *f*_*new *_is created with these genes. This strategy corresponds to the COG approach [23].

• Check the new group against all already existing groups *F *for the following three cases: a) all genes of *f*_*new *_are contained in a group *f*_*i*_. Then *f*_*new *_is not needed and will be removed. b) If the intersection of *f*_*new *_with a group *f*_*i *_is ≥ 3 genes and there are remaining genes in *f*_*new *_not contained in group *f*_*i*_, check these remaining genes for triangle relationships in *f*_*i *_and include them if possible. If all remaining genes could be included in group *f*_*i*_, *f*_*new *_is not needed and will be deleted. c) There exists at least one gene from *f*_*new *_which could not be included in any group *f*_*i *_then *f*_*new *_is added to the set *F*.

• Check all groups of set *F *for subset relationships. Delete the smaller one from *F *and keep only one set in case of equivalence.

Based on this procedure a gene can be part of several functional groups, a functional group can contain several genes from the same genome (inparalogs) but outparalogs are excluded by the *rbm *approach.

#### Determination of gene patterns

As described above, for our approach we define a gene pattern as a set of shared genes within a given genomic neighbourhood. This definition corresponds to a problem known as *gene team model *[29-32], which searches for a set of gene groups that co-occur in a given set of genomes. Further information on formal models can be found in chapter 8 of Mandoiu [33]. The order and the orientation of the genes need not be conserved, and insertions/deletions are allowed within the gene patterns. For in depth analysis we use the concept of pattern instance describing these properties, which are neglected in the process of pattern determination. The approaches mentioned above [29-32] are different with respect to the following features: i) if they are designed for two or more input genomes, ii) if they restrict a gene to be unique in a genome/chromosome or if paralogs are allowed. In addition, these approaches require consistent family assignments of genes for all input genomes, which is not available or incomplete in many cases. Hu and colleagues [34] call this type of problem *gene pattern mining problem *and describe a very similar approach compared to ours.

For the pattern discovery step we have implemented two methods: a systematic search and a heuristic to reduce the search space. The systematic search is adapted from the character enumeration approach successfully applied in motif search algorithms like Pratt [35,36] and TEIRESIAS [37] with the difference that the basic unit to enumerate is a functional group instead a single character.

Given the set of functional groups *F *of the previous step ordered by a numerical identifier and the parameters *minPatternLength *and *q *(quorum) describing the minimal length of a pattern as well as the minimal number of different sequences in which a pattern should be present, the systematic search is carried out as follows:

Let *P *denote the set of pattern to be determined, then *P *is initialized with patterns of length 1 represented by the entities *f *∈ *F*. Each pattern is associated with a set of (meta-)genome identifiers *G*_*p *_⊂ *G *where it occurs. In each iteration *i*, *i *= 2, ..., |*F*| all patterns *p *∈ *P *∧ |*p*| = *i*-1 (the patterns from the last iteration having length *i*-1) are enlarged by the functional group having the next higher identifier and checked whether the corresponding set of genome identifiers covers more or equal entities than *q*. If yes, the pattern will be added to *P*. This systematic search guarantees to find all patterns fulfilling the constraints of the given parameters, but it has an exponential growing search space depending on the number of functional groups $|F|:O\left(\sum_{i=1}^{\left|F\right|}\left(\begin{array}{c} \left|F\right|\\ i \end{array}\right)\right)$

Therefore, a second method was implemented combining the systematic search and a heuristic. In order to generate a pattern there are two entities, which have to be checked: i) the functional groups as constituents for a pattern and ii) the (meta-) genome(s), where the pattern is present. In contrast to the systematic search based on the functional groups the heuristic inverts the constituents and the test in the following sense. First the gene patterns are generated as described above until a user-specified length *minLengthHeuristics *with default value of five. Second, for the set *P *of patterns found so far, the associated sets of (meta-)genome identifiers *G*_*p *_are collected and filtered to be redundancy-free. The resulting set contains all genome combinations *G*_*p*_, where a pattern can occur. Then, for each (meta-)genome combination *G*_*p *_the largest set of shared functional groups are determined applying the intersection operation. Given a set of genome combinations the advantage of this heuristic is its ability to detect long gene patterns very quickly without explicit generation and testing of all functional group combinations, which can be a huge number.

### System architecture

The system was implemented using a three-tier architecture that allows flexibility for subsequent integration of MetaMine into other systems. It can be used in two modes: as stand-alone system with direct access to a local database or as a client-server application using web services for all database operations.

The persistence layer is responsible for the permanent storage and retrieval of all necessary data for MetaMine. Therefore, it provides storage and retrieval functions for the MEGDB. In addition, there are functions to read and write to the file system especially for the import of key gene sequences stored in FASTA format and to import and export the analysis results as XML files for further data exchange. The BLAST database file containing the protein sequences for the BLAST searches belongs also to this layer. In principle it is possible to exchange the underlying database with an own version.

The application layer contains all objects and methods implementing the application logic by providing methods for all functions provided to the user (see description of prototype for details). In addition, interfaces to external programs like BLAST for the sequence similarity search exist, as well as readers and writers for specific file formats used in molecular biological applications.

The presentation layer comprises the graphical user interface and the controller which activates the functions chosen by the user.

## Results and discussion

The MetaMine software was tested using the MEGDB which contains high-quality georeferenced marine genomes and metagenomes of prokaryotic origin. This pattern detection approach can also successfully applied on large metagenome data sets based on shot-gun sequencing approaches as long as a significant fraction contains sequences with at least two predicted genes. Harrington *et al. *[4] reported recently that 47% of short metagenome sequences obtained by whole genome shot-gun sequencing in fact have neighbours even in the same transcriptional direction.

The focus of MetaMine is searching for gene patterns representing biological functions occurring in specific environmental contexts regardless their evolutionary history. Even if the current public DNA sequence databases covers only a fraction of the natural prokaryotic diversity [38], numerous environmentally relevant microbial pathways occurring in marine environments have been discovered and genetically described [39-41]. Two examples illustrating the benefits of the semi-automatic gene pattern discovery procedure of MetaMine for the study of globally important metabolic pathways in (meta-)genomic context are presented.

### Archaea C1 metabolism gene patterns

**Figure 2:**
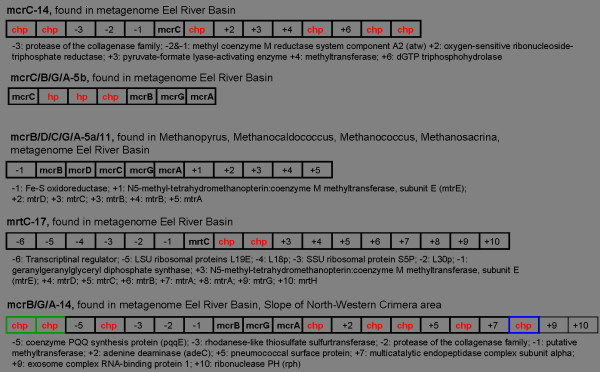
**Consensus patterns**. Five consensus patterns (longest extension with mismatches) of the analysis with mcr gene. Chp represents conserved hypothetical proteins. All BLAST searches were carried out with a threshold of 1E-5.

Methanogenesis and the Anaerobic Oxidation of Methane (AOM) are two microbial metabolic pathways of environmental relevance, because they produce and consume the greenhouse gas methane in marine sediments, respectively. A MetaMine analysis using archaeal C1 key genes (mcrA, mcrB, mcrC, mcrD, mcrG, mrtC taken from [42]) discovered five distinct gene patterns called mcrB/G/A-14, mcrC-14, mcrB/D/C/G/A-5a/11, mcrC/B/G/A-5b and mrtC-17, where the name describes the key genes contained and the length of the patterns (see Fig. 2 and Additional file 1). As expected, the analysis shows that all key genes and their associated patterns occur exclusively in the habitat type "sediment". The computational results foster the functional coupling of the genes with respect to their involvement in the C1 metabolism. Furthermore, all patterns, except that found in isolated organisms (mcrB/D/C/G/A-5a/11), revealed conserved hypothetical genes (chp; all red genes in Fig. 2) indicating their potential role in these particular metabolisms. These genes represent interesting new functional candidates and should be prior targets for wet-lab experiments. Moreover, four of the five gene patterns could be detected on metagenomic fragments, but not on complete genomes, which might reflect the specific modifications needed for the AOM metabolism as compared to the classical methanogenesis pathway [43,42,39].

### Carbon monoxide oxidation gene patterns

Carbon monoxide (CO) is a gas evaporating from the ocean into the atmosphere. CO reacts with hydroxyl radicals who are also able to oxidize methane and nitrous oxides and is therefore an indirect mediator of the greenhouse effect [40]. Interestingly, microorganisms from the surface ocean water have recently been shown to carry genes encoding CO oxidation pathways, potentially influencing the diffusion of this gas in the atmosphere [44,45].

**Figure 3:**
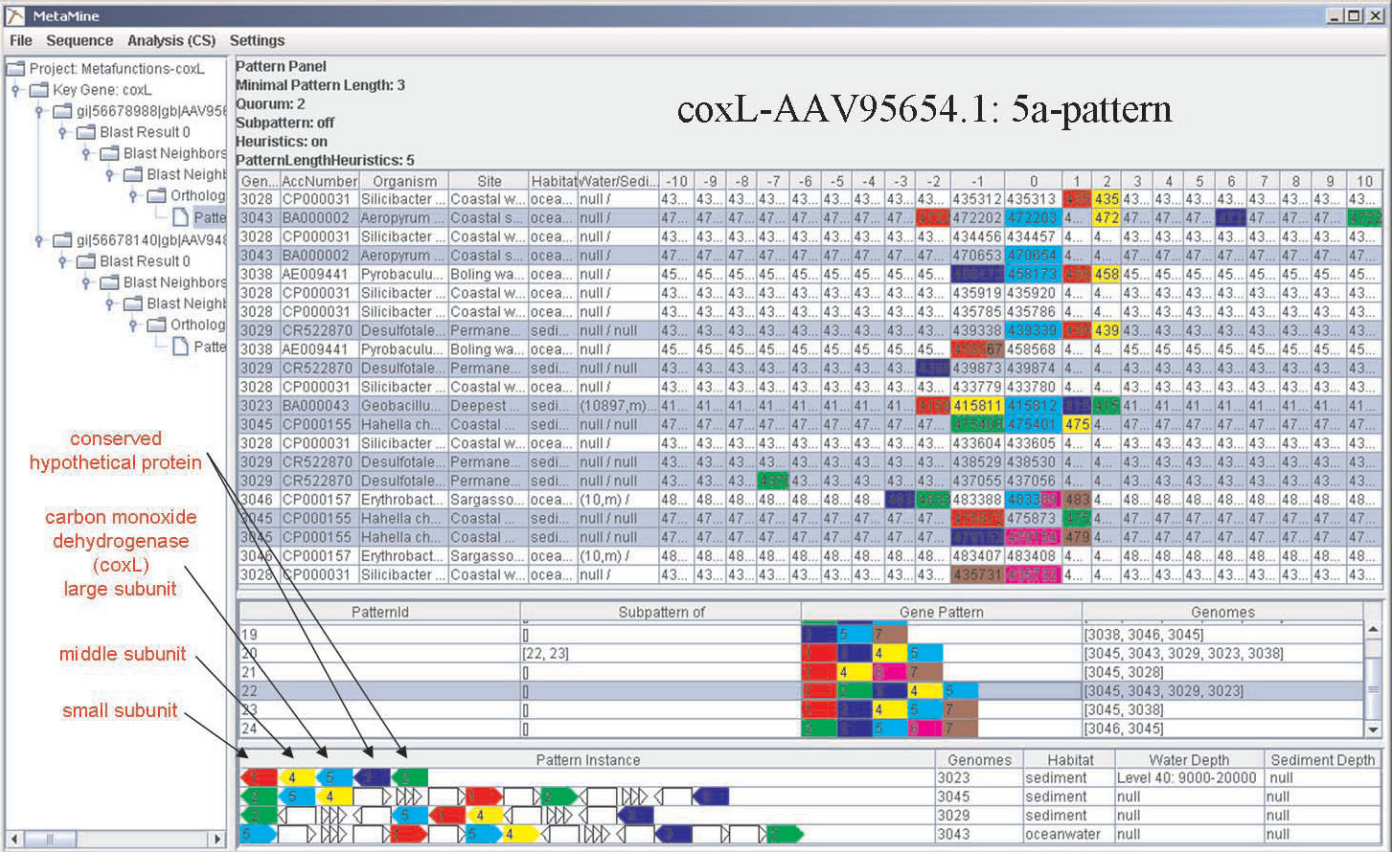
**Analysis of coxL**. All BLAST searches were carried out with threshold 1E-1.

In order to search for gene patterns associated with CO oxidation, the corresponding key genes have been used as input for a MetaMine analysis (coxL, GenBank: AAV95654 and GenBank: AAV94806[44,40]). The results show four main gene patterns including up to five genes (Fig. 3 and Additional file 1). Two conserved hypothetical genes can be found within these patterns, which designate them as potentially relevant for the CO oxidation pathways (Fig. 3, green and blue genes). Furthermore, one out of the eight gene patterns could be found exclusively in genomes isolated from marine sediments/geothermal sources, but not in genomes originating from the water column (Fig 3, pattern ID 70).

## Conclusion

The exponentially growing DNA sequence datasets can only be handled effectively using semi-automatic processing pipelines that go beyond similarity based approaches. It has been shown that comparative approaches can significantly improve the quantity and quality of functional assignments leading to deeper insights into the complex metabolic and regulatory processes in a cell. The ecogenomic revolution initiated by the Venter cruises some years ago has opened the door to expand this approach by correlating syntenic gene patterns with specific environmental parameters and associated prevailing biological processes.

MetaMine offers a targeted, knowledge driven system to detect gene patterns for subsequent correlation with environmental information. First, the system is meant to confirm existing biological knowledge about genes involved in specific processes or pathways. Second, the approach has the potential to detect genes of so far unknown functions but functionally linked to specific habitat parameters. By integrating structural genomic information with environmental conditions MetaMine helps to find the "needle in the (meta)genomic haystack" especially for genes of so far unknown function. This reduced set of genes contains than prime candidates for further detailed functional analysis in the wet-lab. A use-case for *Archaea *C1 metabolism and CO oxidation genes showed that meaningful initial results can be quickly generated using MetaMine and a set of user-defined key genes. Further developments will concentrate on the incorporation of further genomic and metagenomic sequences, additional environmental parameters and further methods for the detection of functionally equivalent genes. In addition, to enhance the usability of MetaMine we plan to include links to external resources like GO or KEGG to support functional annotation as well as concepts to compare the functional groups found by MetaMine with other systems like COG.

## Availability and requirements

Project name: MetaMine

Project home page: 

Operating systems: Every OS with Java JRE 1.5 or higher (tested on Windows/Linux).

Programming language: Java.

Other requirements: Java JRE 1.5 or higher

License: license-free.

Any restrictions to use by non-academics: MetaMine may not be sold or bundled with any type of commercial application.

## Abbreviations

AOM: Anaerobic Oxidation of Methane; CO: carbon monoxide; coxL: carbon monoxide dehydrogenase (large subunit) gene; mcr/Mcr: methyl-coenzyme-M reductase gene/protein; rbm: reciprocal best match; COG: clusters of orthologous groups of genes; GUI: graphical user interface; XML: extensible markup language; FP6: the sixth framework programme of the European Union; NEST: new and emerging science and technology

## Authors' contributions

UB designed and implemented MetaMine and drafted the manuscript. RK designed and implemented the current version of the underlying database and integrated the metagenomic data. UB and TL carried out and evaluated the biological test examples. UB, RK and TL participated in installing the MetaMine system as client server version at MPI. FOG is leading the EU-project MetaFunctions, gave advises for software development and has made revisions and contributions to the manuscript.

## Supplementary Material

Additional file 1 **The file contains screenshots of the analyses of the two examples and a more detailed description of the corresponding consensus patterns.** Click here for file

Additional file 2 **The file contains a user guide about MetaMine version 1.2.** Click here for file
