# Supplementary material for: MetaMine – A tool to detect and analyse gene patterns in their environmental context
Source: BMC Bioinformatics. 2008 Oct 28;9:459. doi: 10.1186/1471-2105-9-459 (PMC2615450; doi:10.1186/1471-2105-9-459)
Supplement: Additional file 1 — The file contains screenshots of the analyses of the two examples and a more detailed description of the corresponding consensus patterns. [file 1471-2105-9-459-S1.pps]

## Slide 1
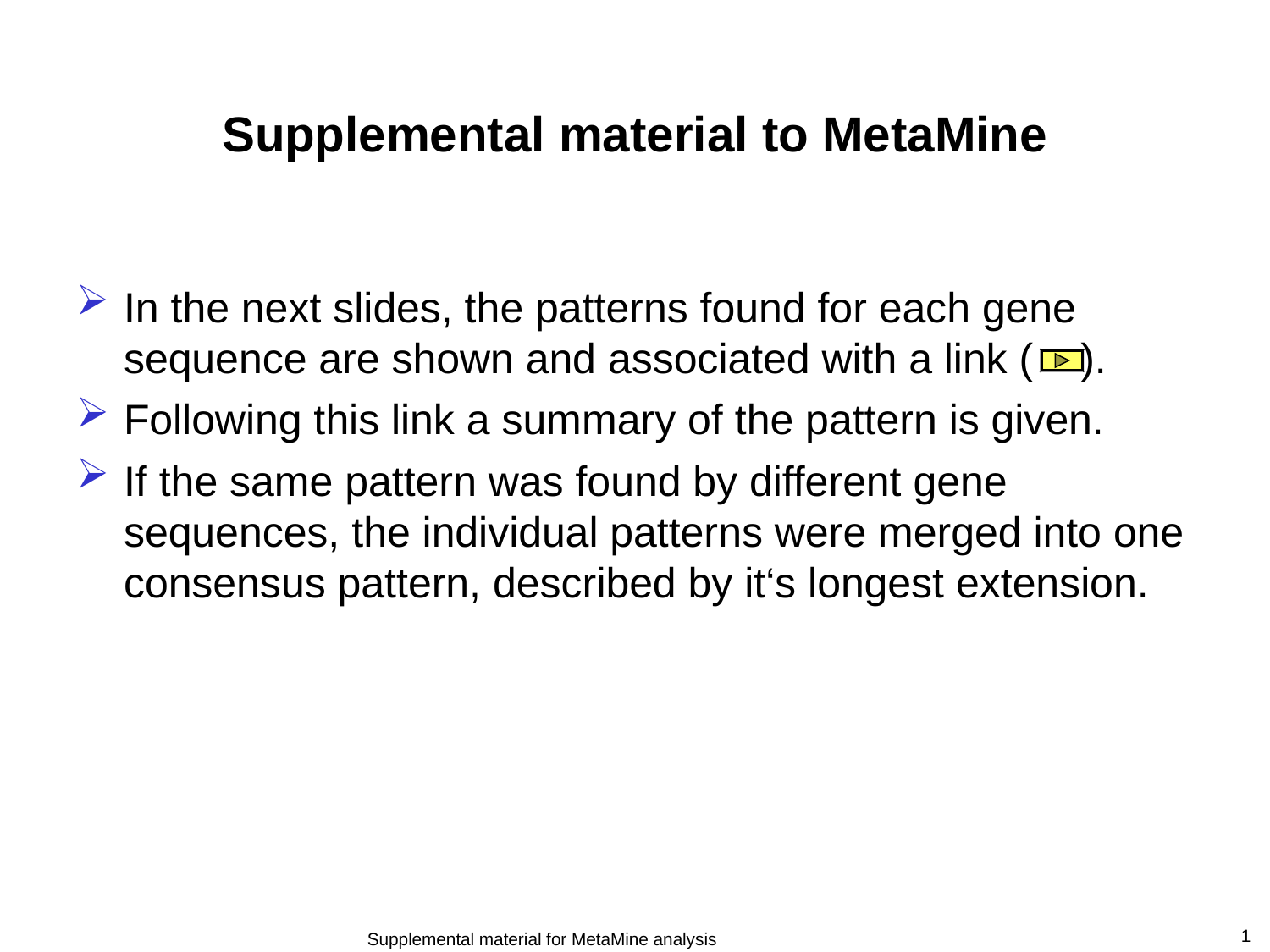

# Supplemental material to MetaMine
In the next slides, the patterns found for each gene sequence are shown and associated with a link ( ).
Following this link a summary of the pattern is given.
If the same pattern was found by different gene sequences, the individual patterns were merged into one consensus pattern, described by it‘s longest extension.
1

## Slide 2
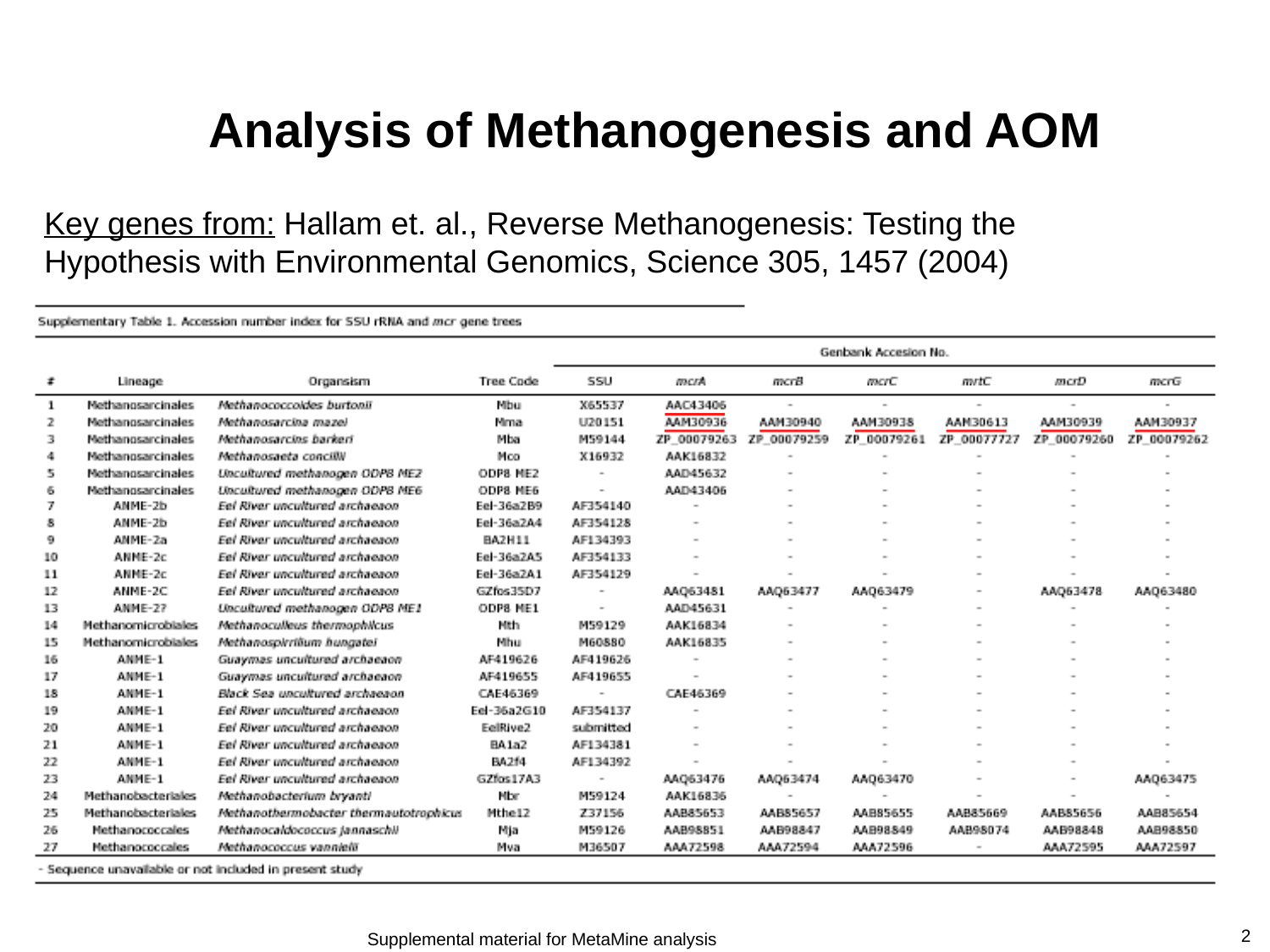

# Analysis of Methanogenesis and AOM
Key genes from: Hallam et. al., Reverse Methanogenesis: Testing the Hypothesis with Environmental Genomics, Science 305, 1457 (2004)
2

## Slide 3
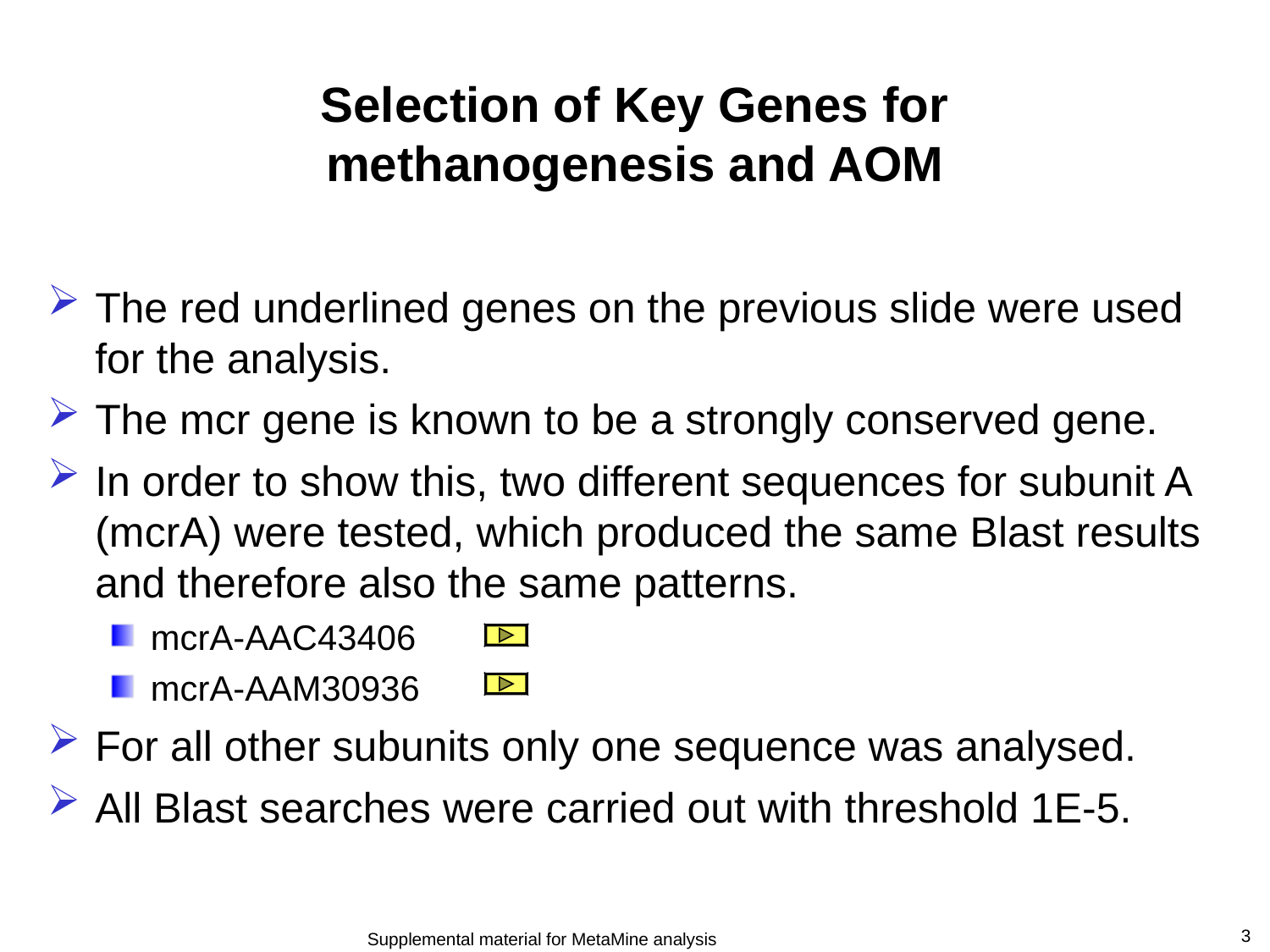

# Selection of Key Genes for methanogenesis and AOM
The red underlined genes on the previous slide were used for the analysis.
The mcr gene is known to be a strongly conserved gene.
In order to show this, two different sequences for subunit A (mcrA) were tested, which produced the same Blast results and therefore also the same patterns.
mcrA-AAC43406
mcrA-AAM30936
For all other subunits only one sequence was analysed.
All Blast searches were carried out with threshold 1E-5.
3

## Slide 4
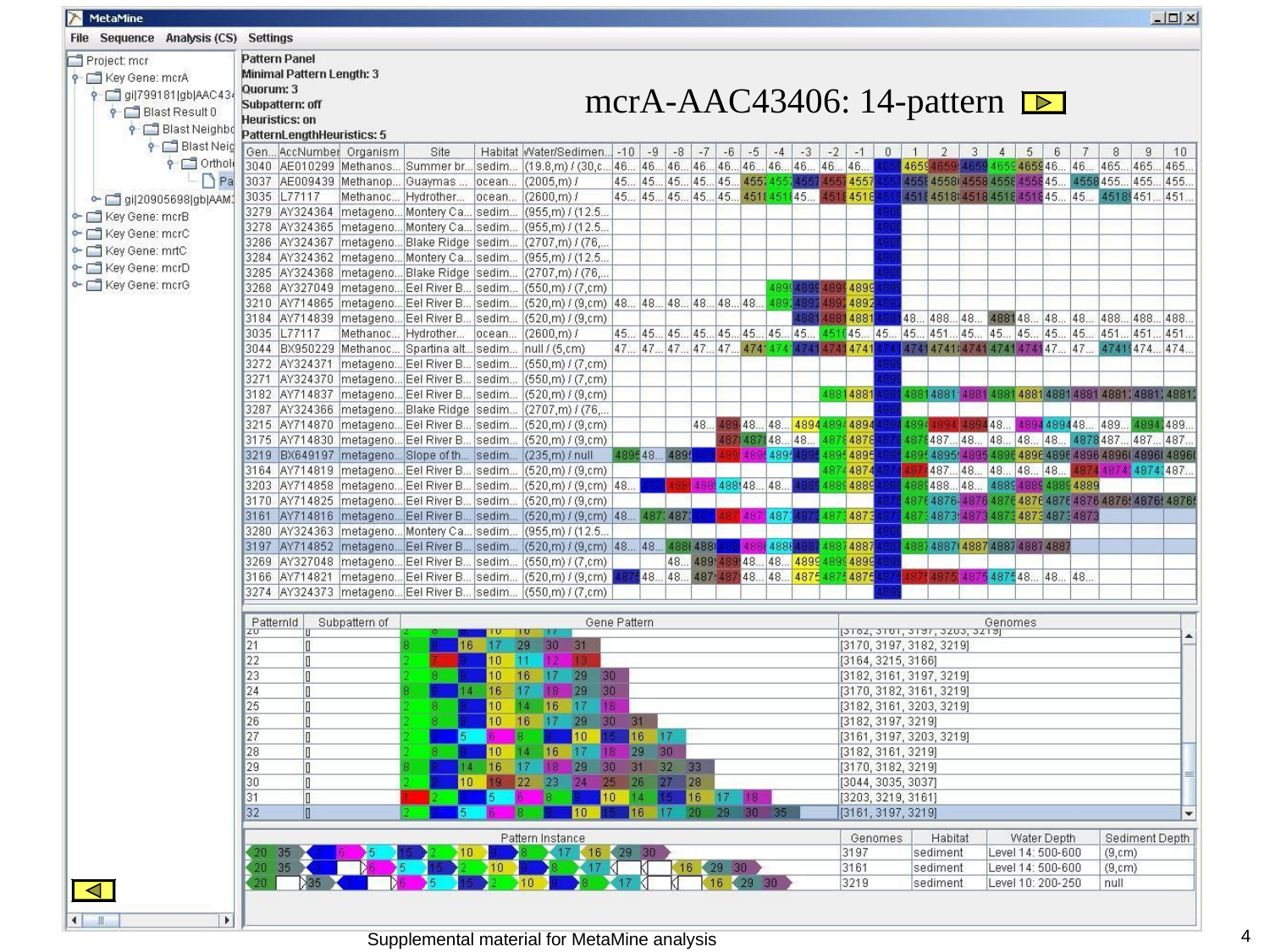

mcrA-AAC43406: 14-pattern
4

## Slide 5
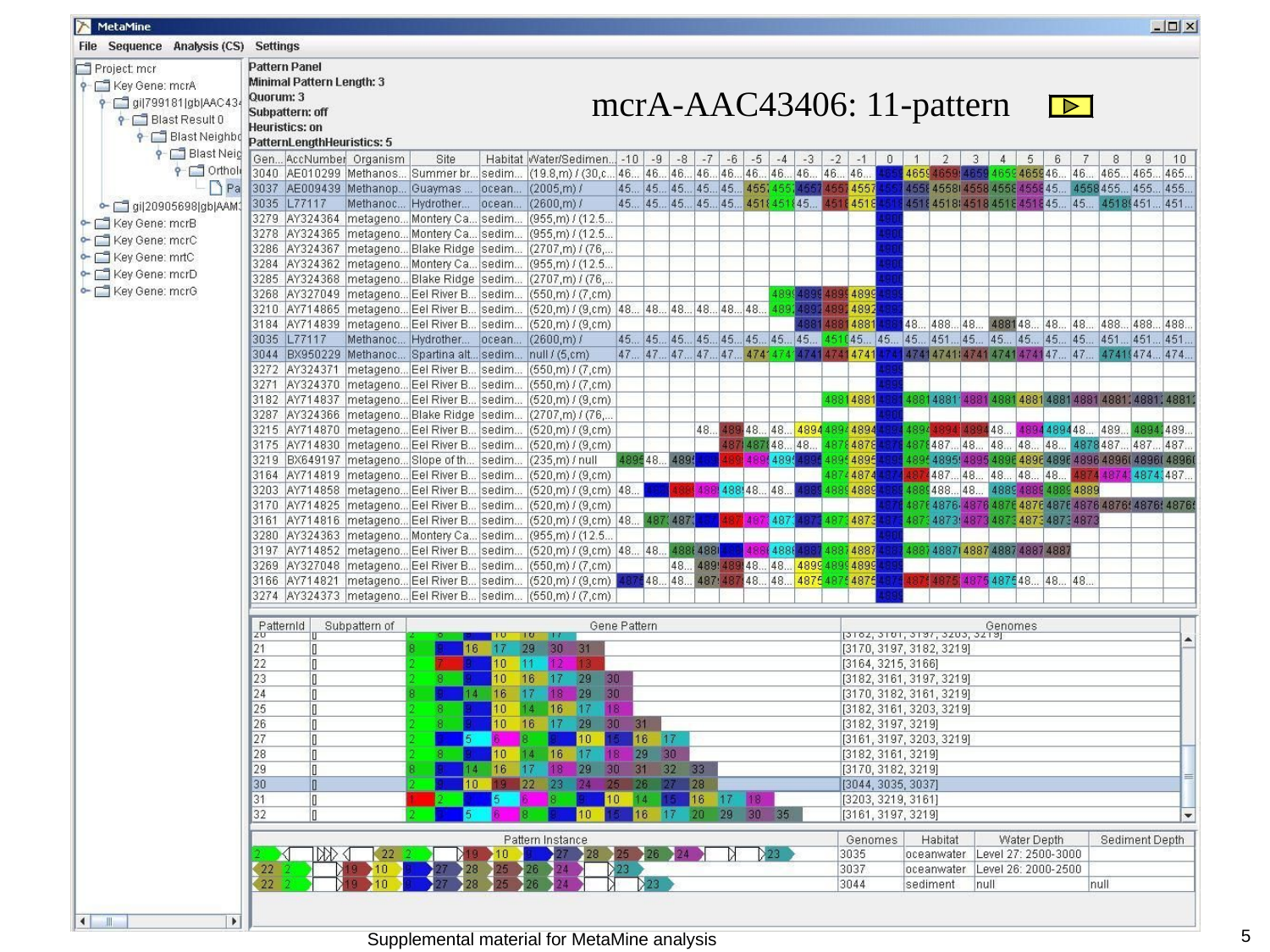

mcrA-AAC43406: 11-pattern
5

## Slide 6
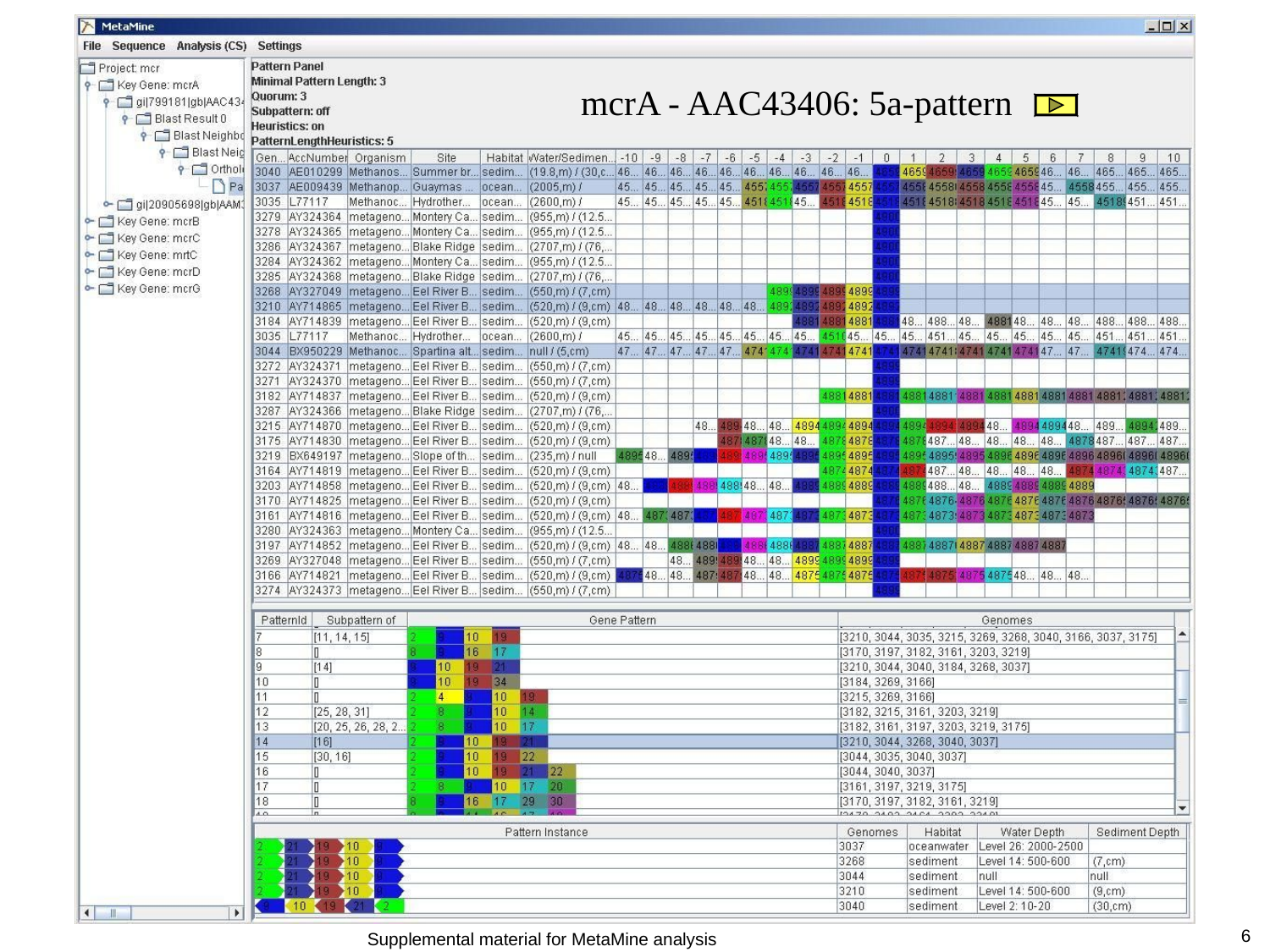

mcrA - AAC43406: 5a-pattern
6

## Slide 7
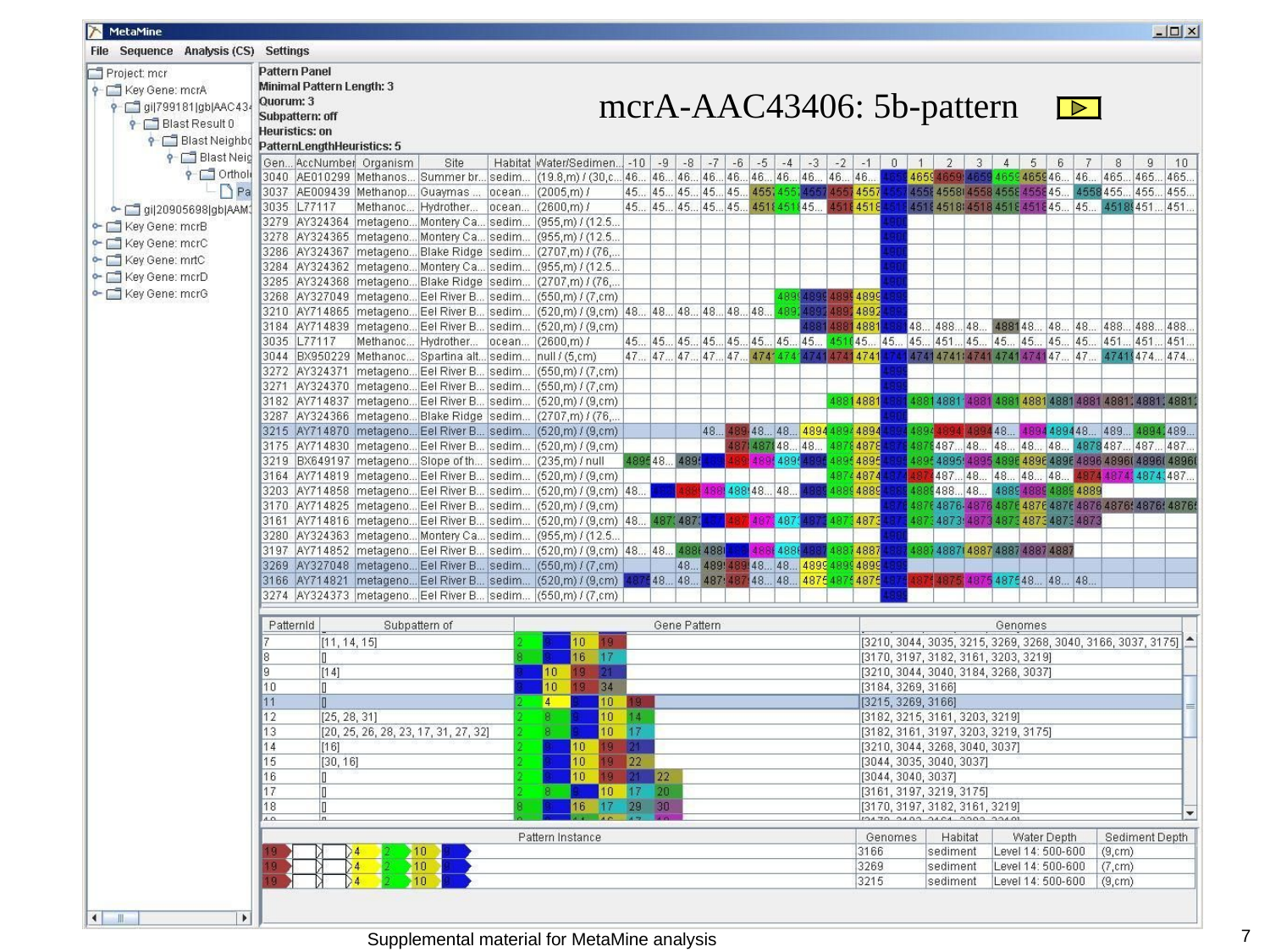

mcrA-AAC43406: 5b-pattern
7

## Slide 8
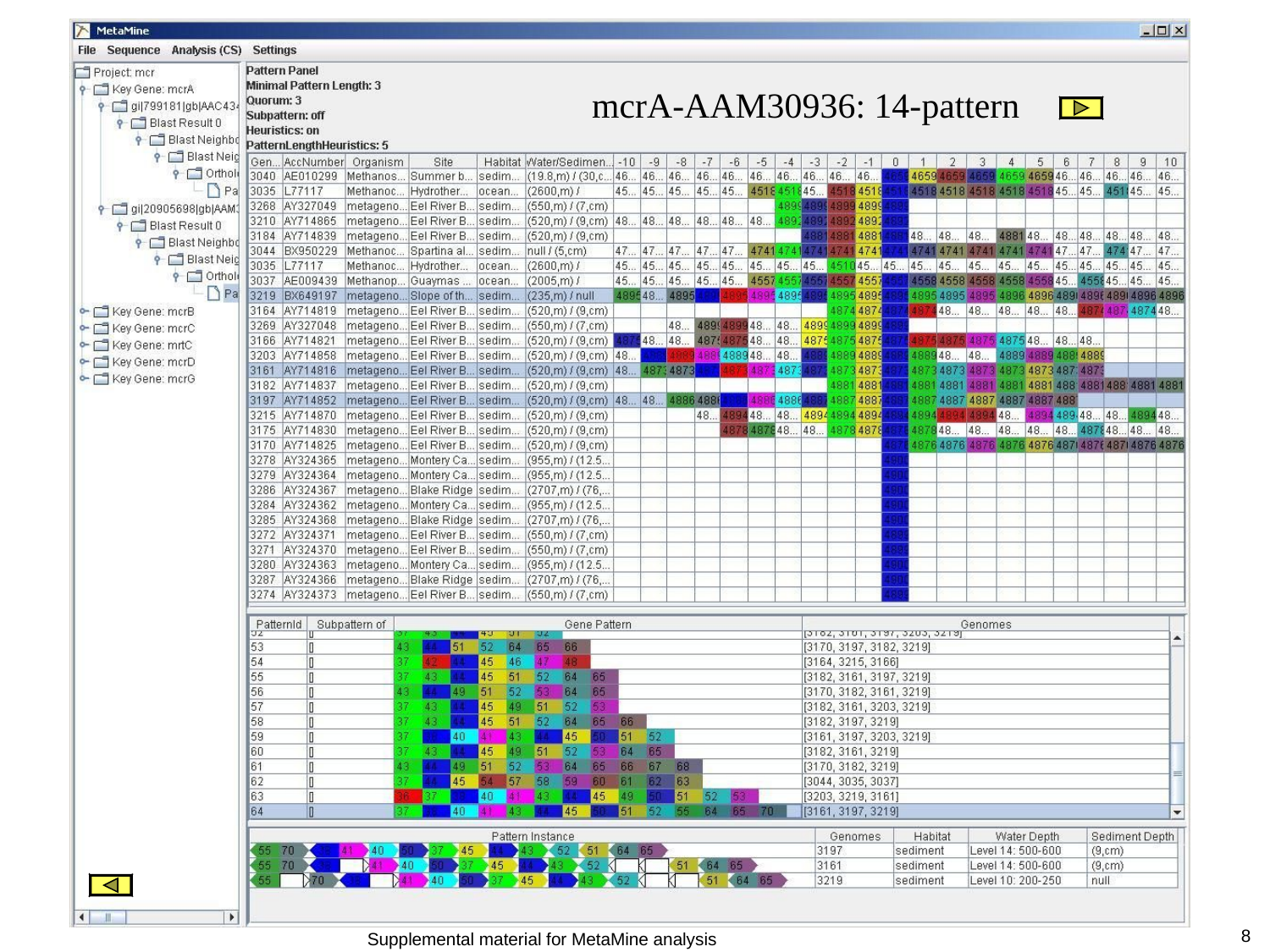

mcrA-AAM30936: 14-pattern
8

## Slide 9
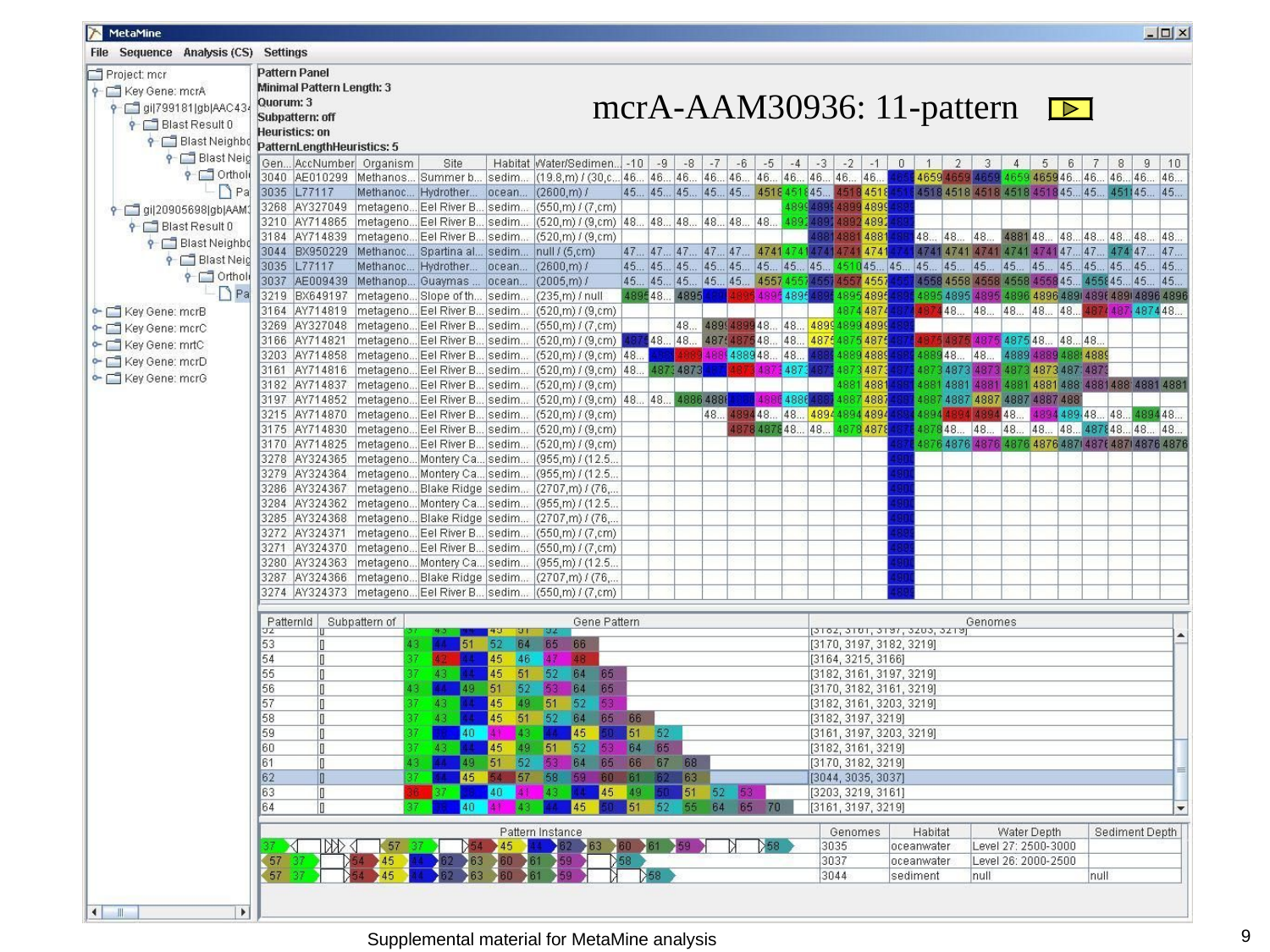

mcrA-AAM30936: 11-pattern
9

## Slide 10
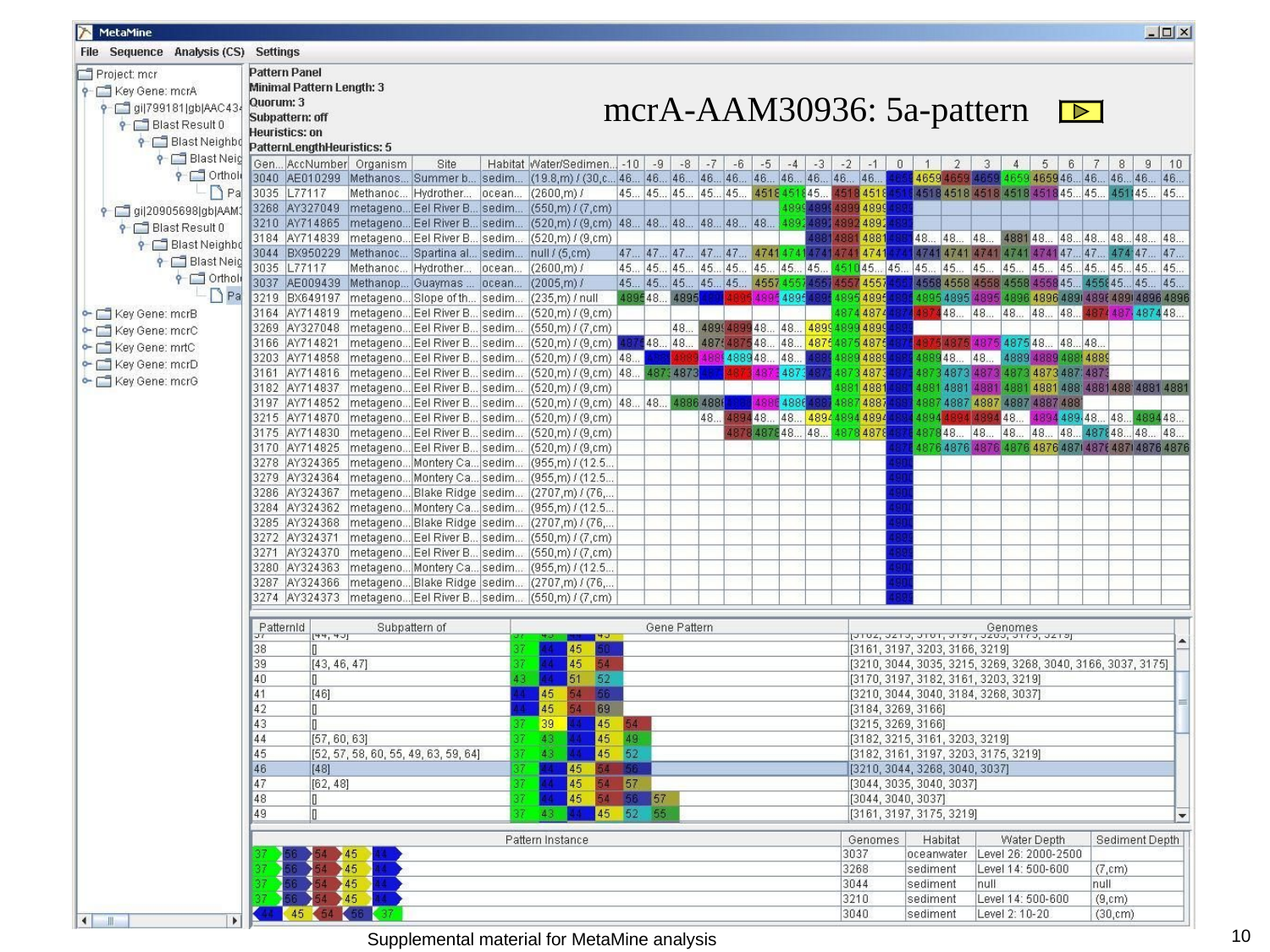

mcrA-AAM30936: 5a-pattern
10

## Slide 11
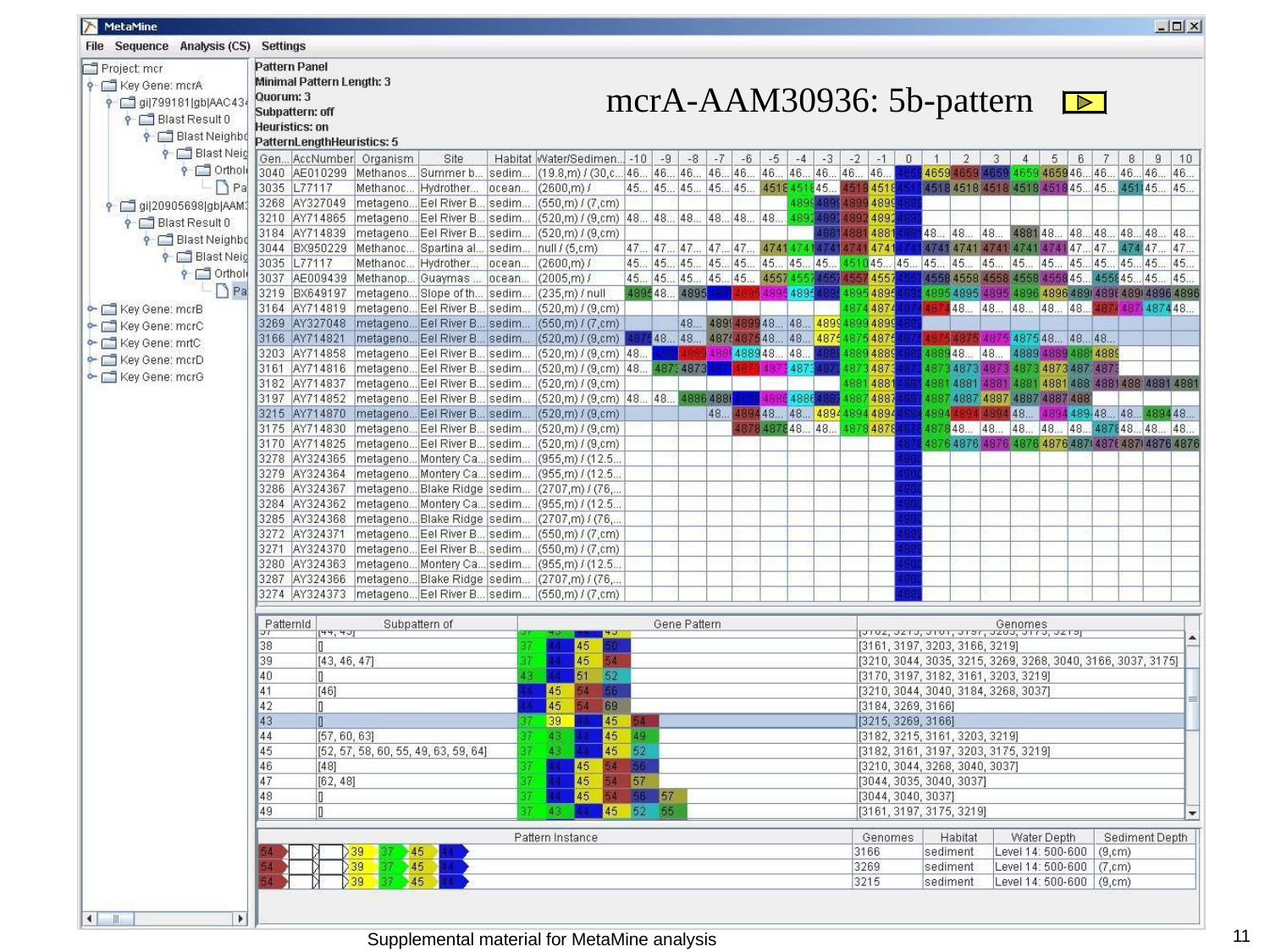

mcrA-AAM30936: 5b-pattern
11

## Slide 12
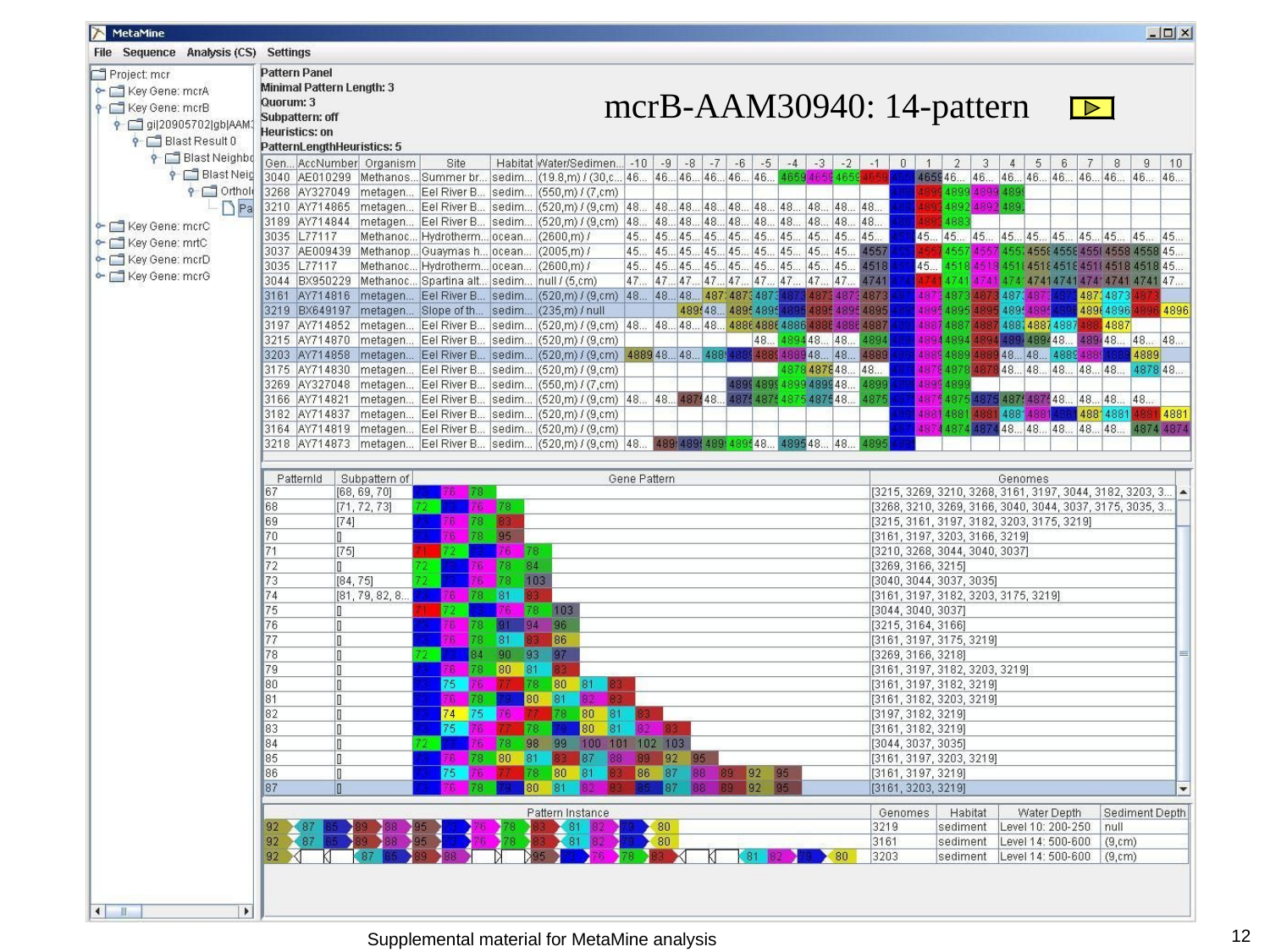

mcrB-AAM30940: 14-pattern
12

## Slide 13
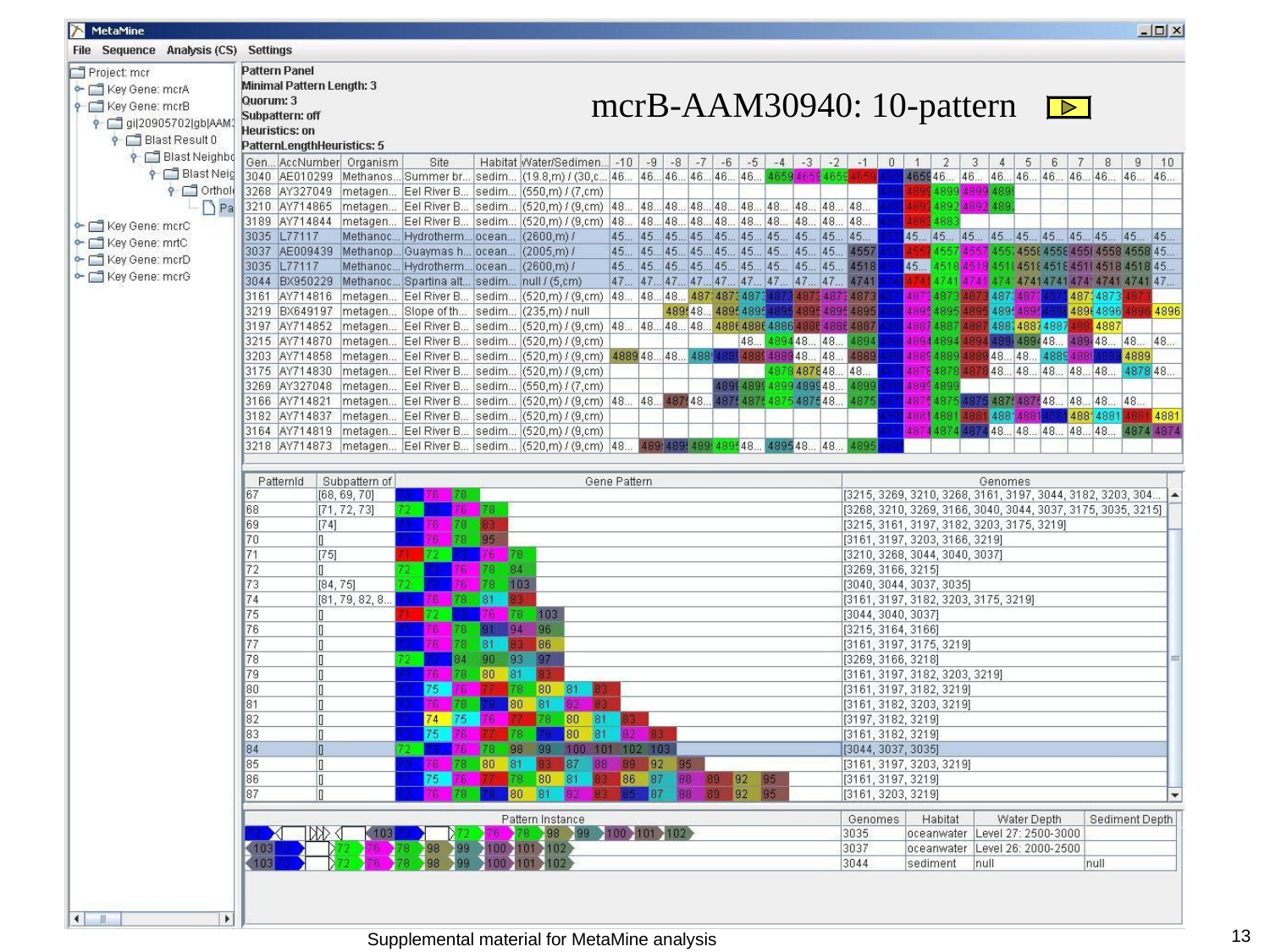

mcrB-AAM30940: 10-pattern
13

## Slide 14
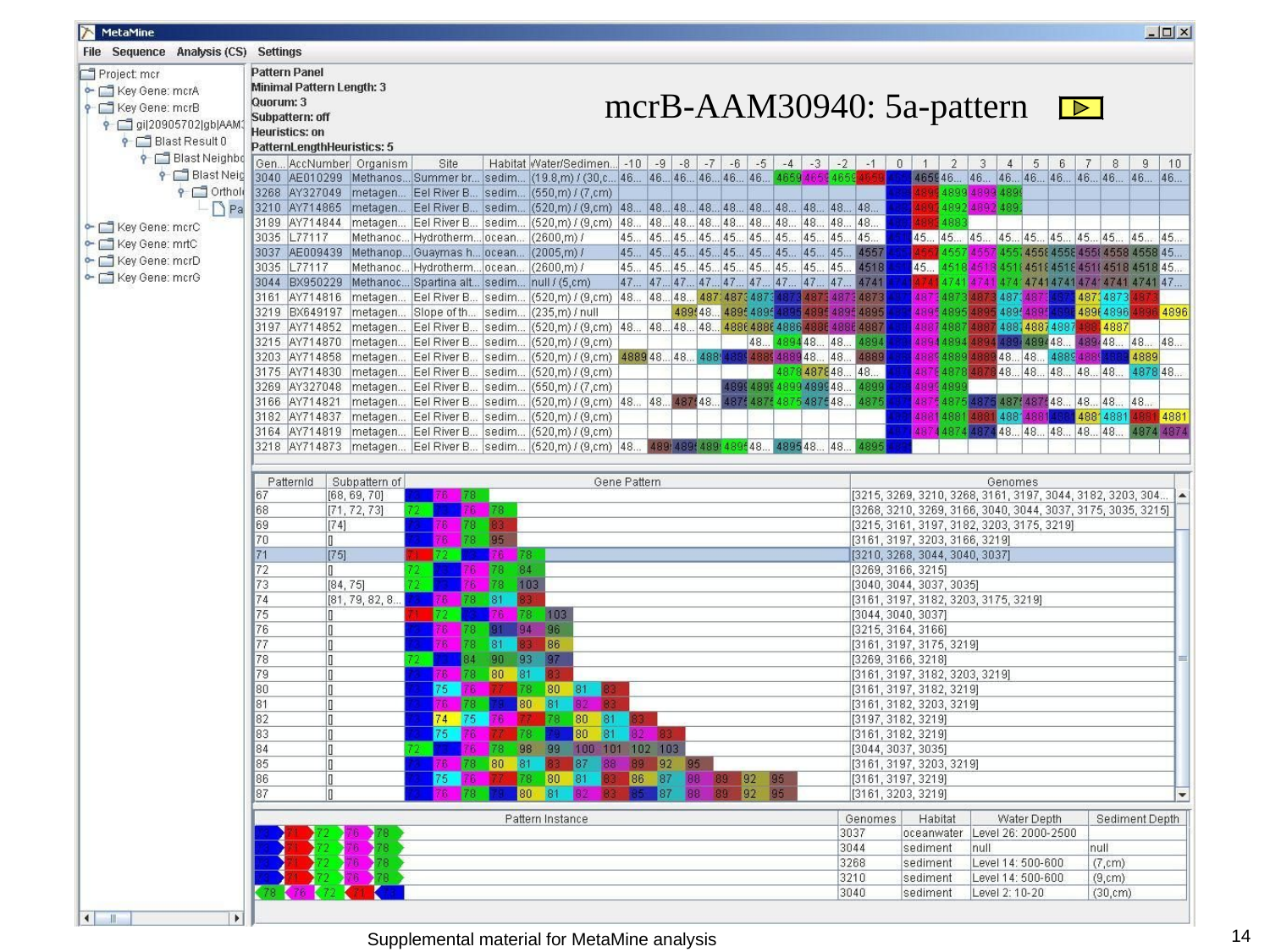

mcrB-AAM30940: 5a-pattern
14

## Slide 15
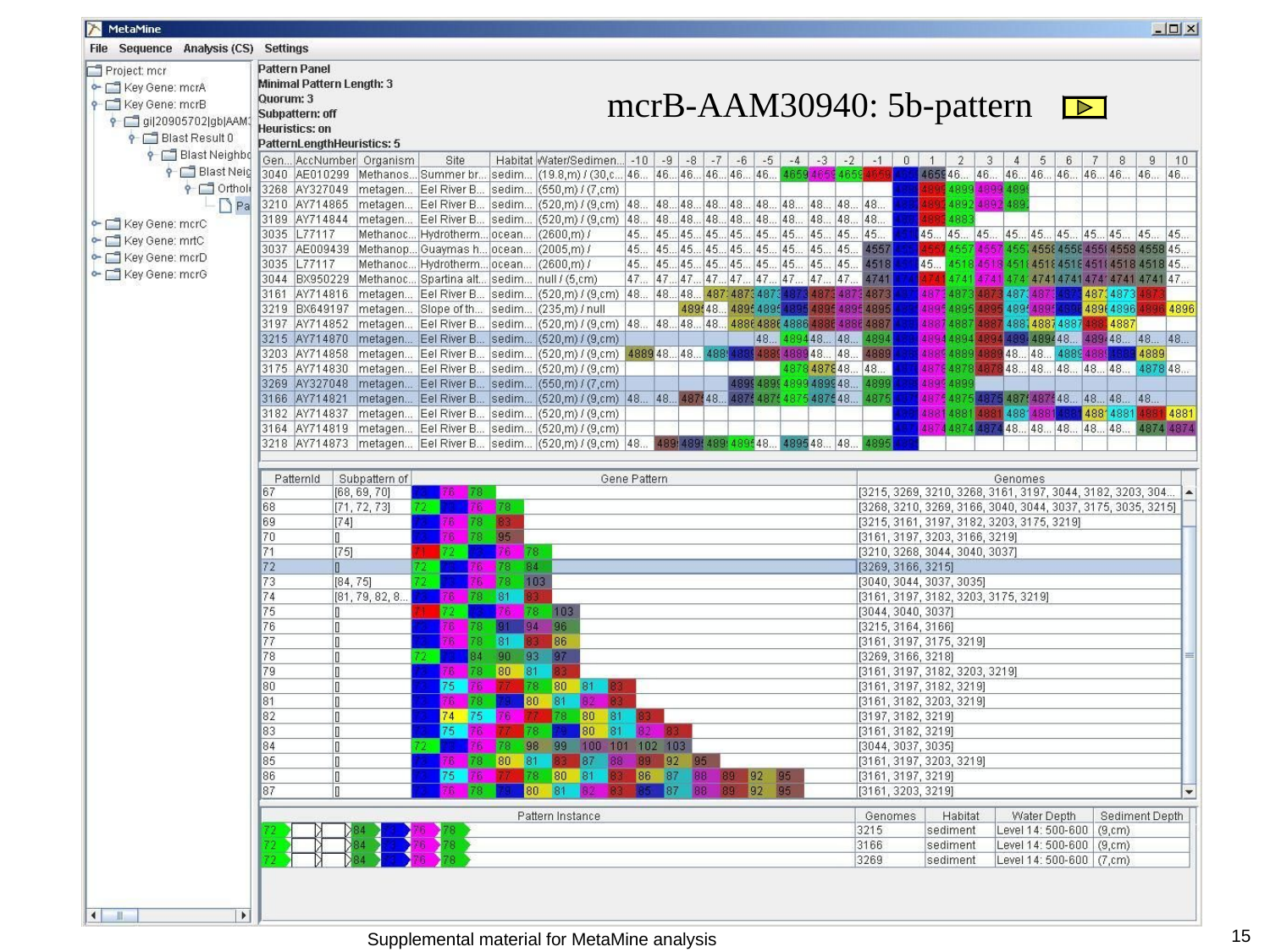

mcrB-AAM30940: 5b-pattern
15

## Slide 16
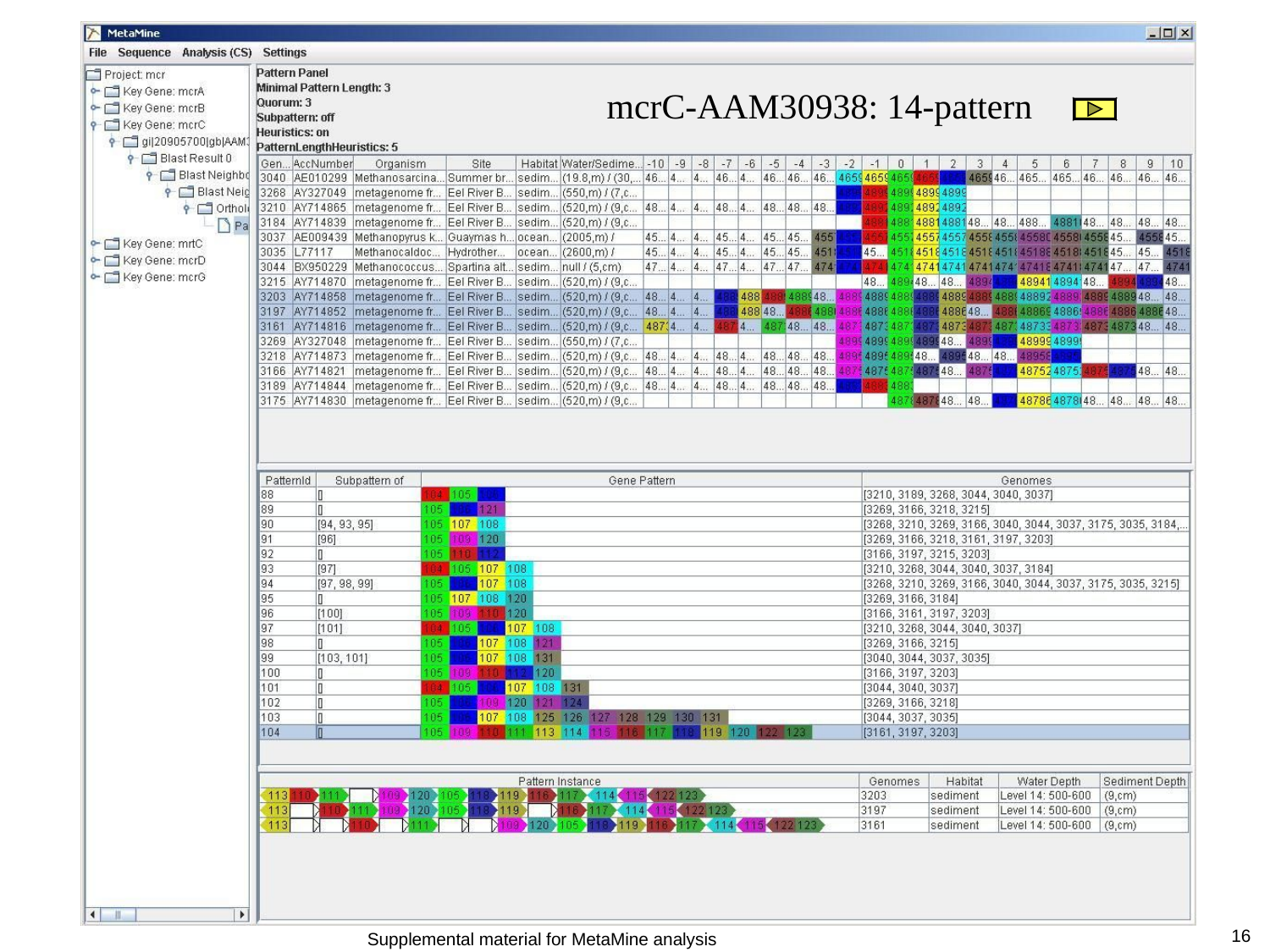

mcrC-AAM30938: 14-pattern
16

## Slide 17
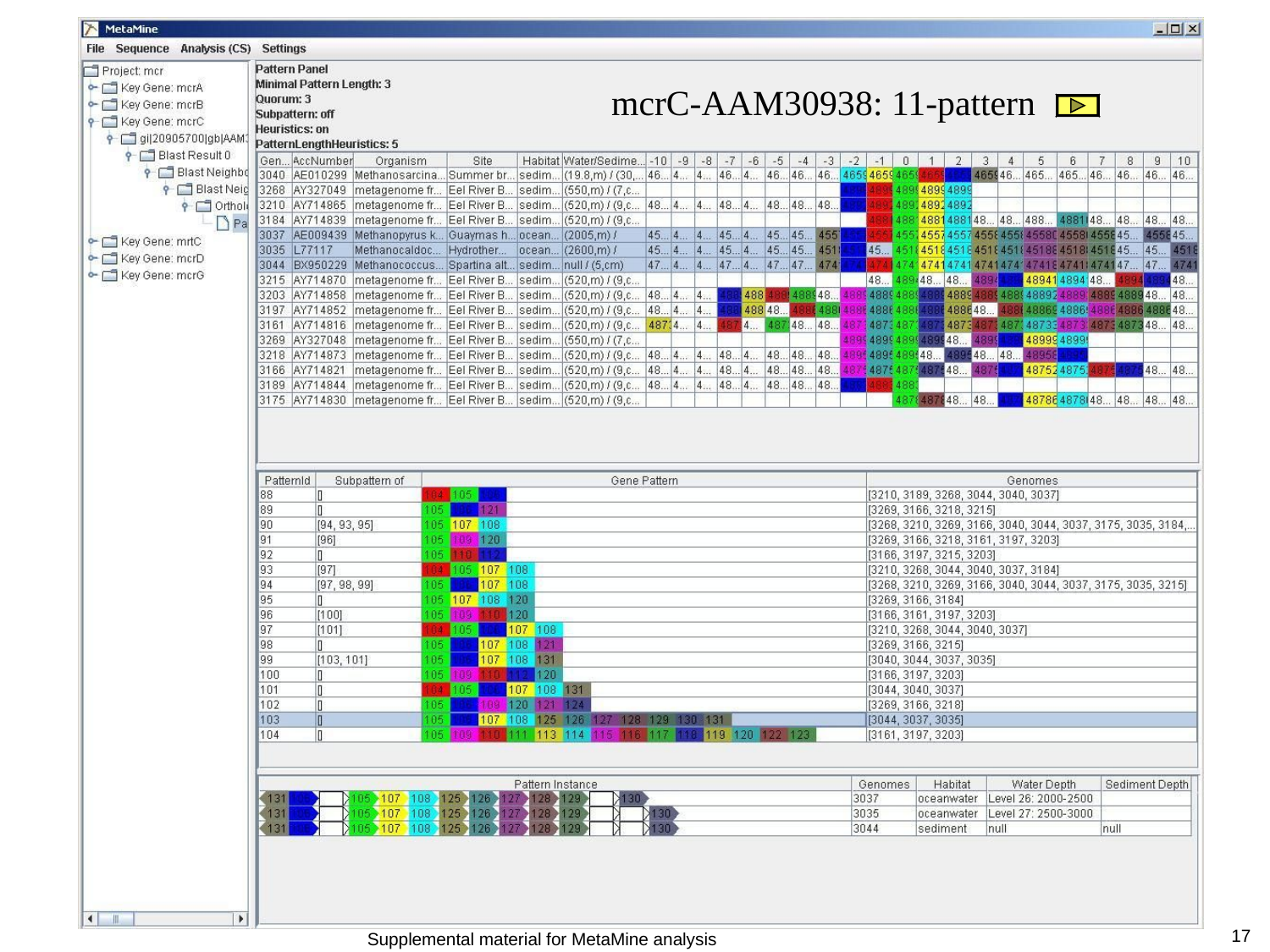

mcrC-AAM30938: 11-pattern
17

## Slide 18
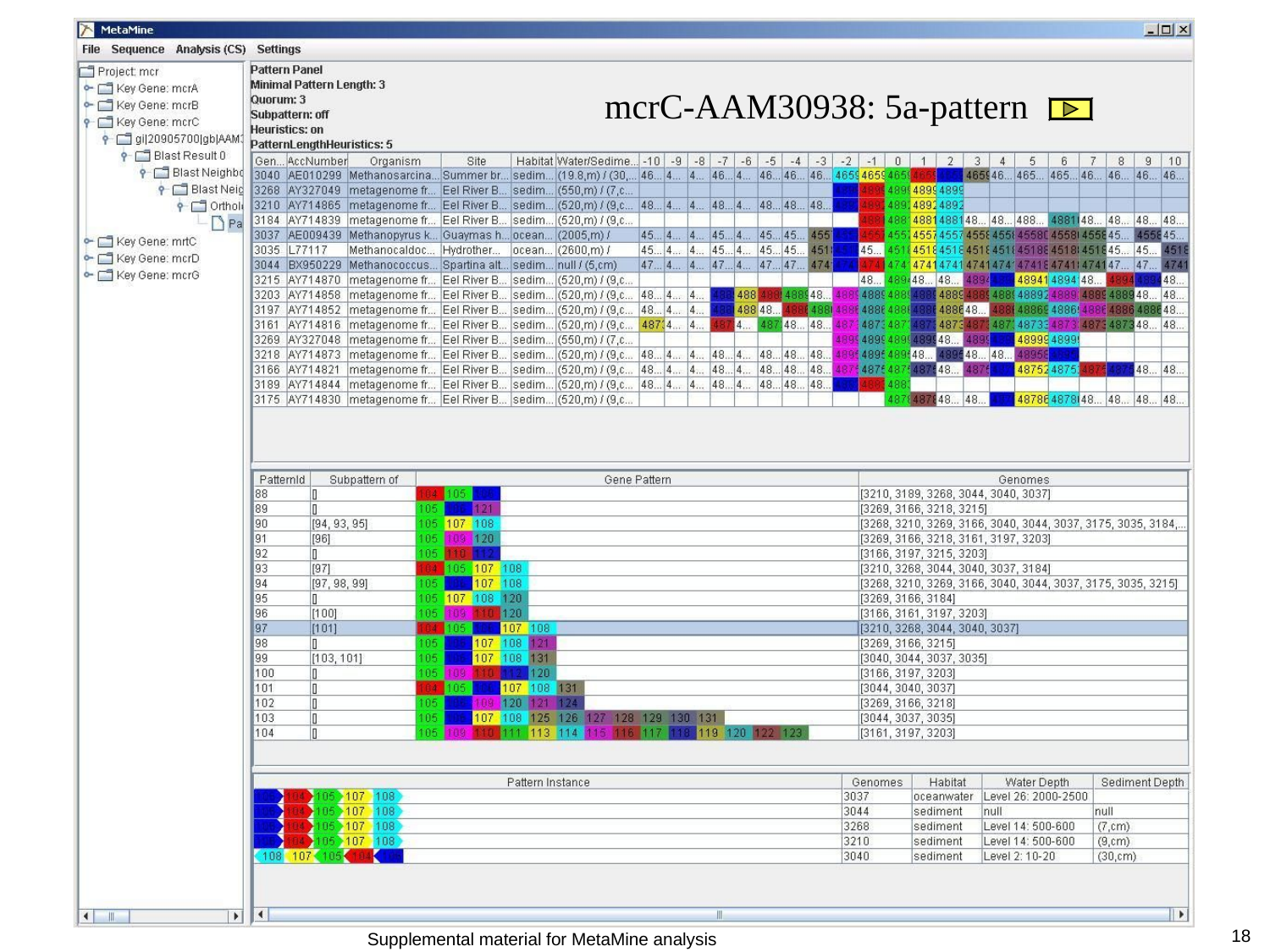

mcrC-AAM30938: 5a-pattern
18

## Slide 19
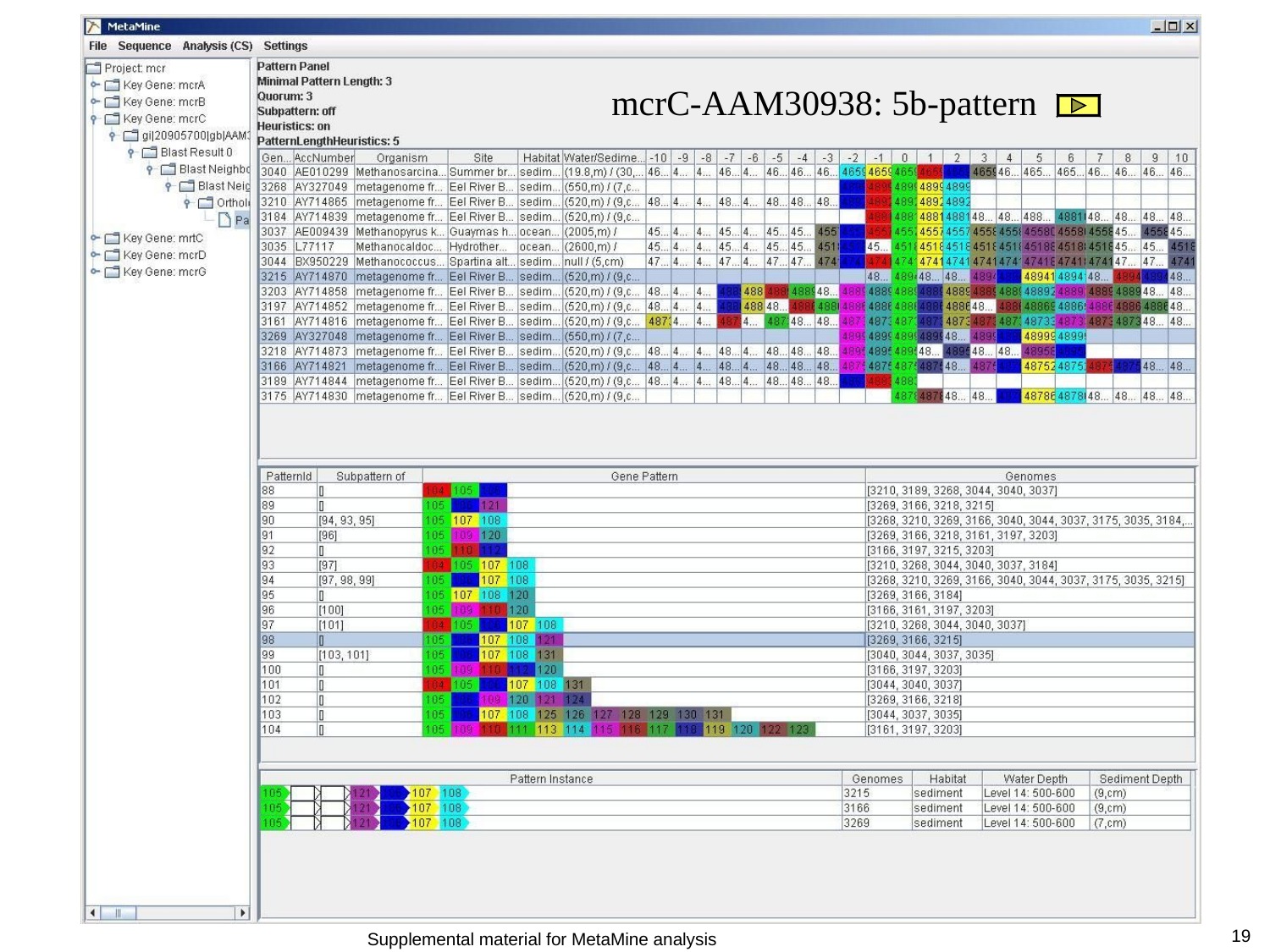

mcrC-AAM30938: 5b-pattern
19

## Slide 20
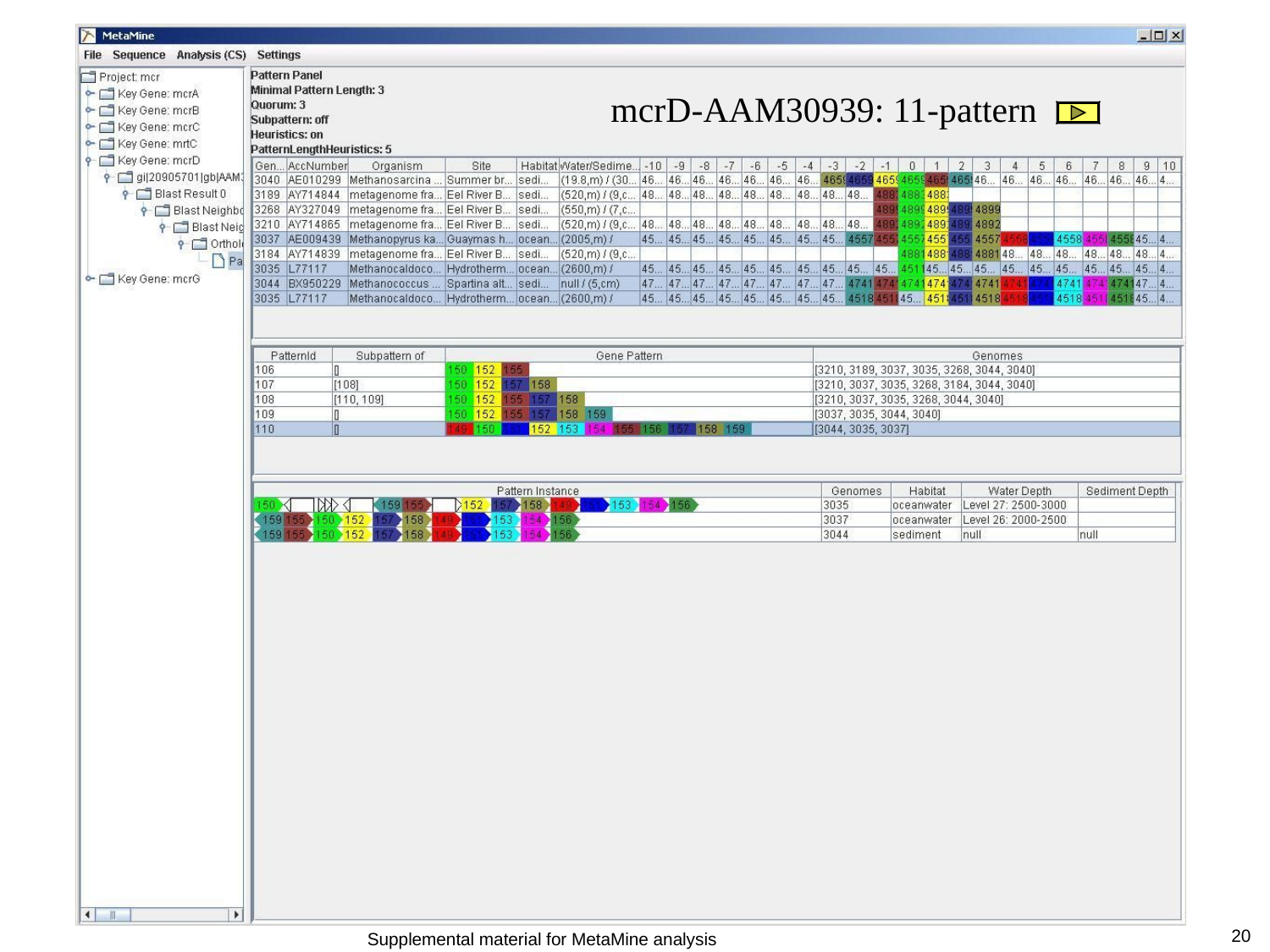

mcrD-AAM30939: 11-pattern
20

## Slide 21
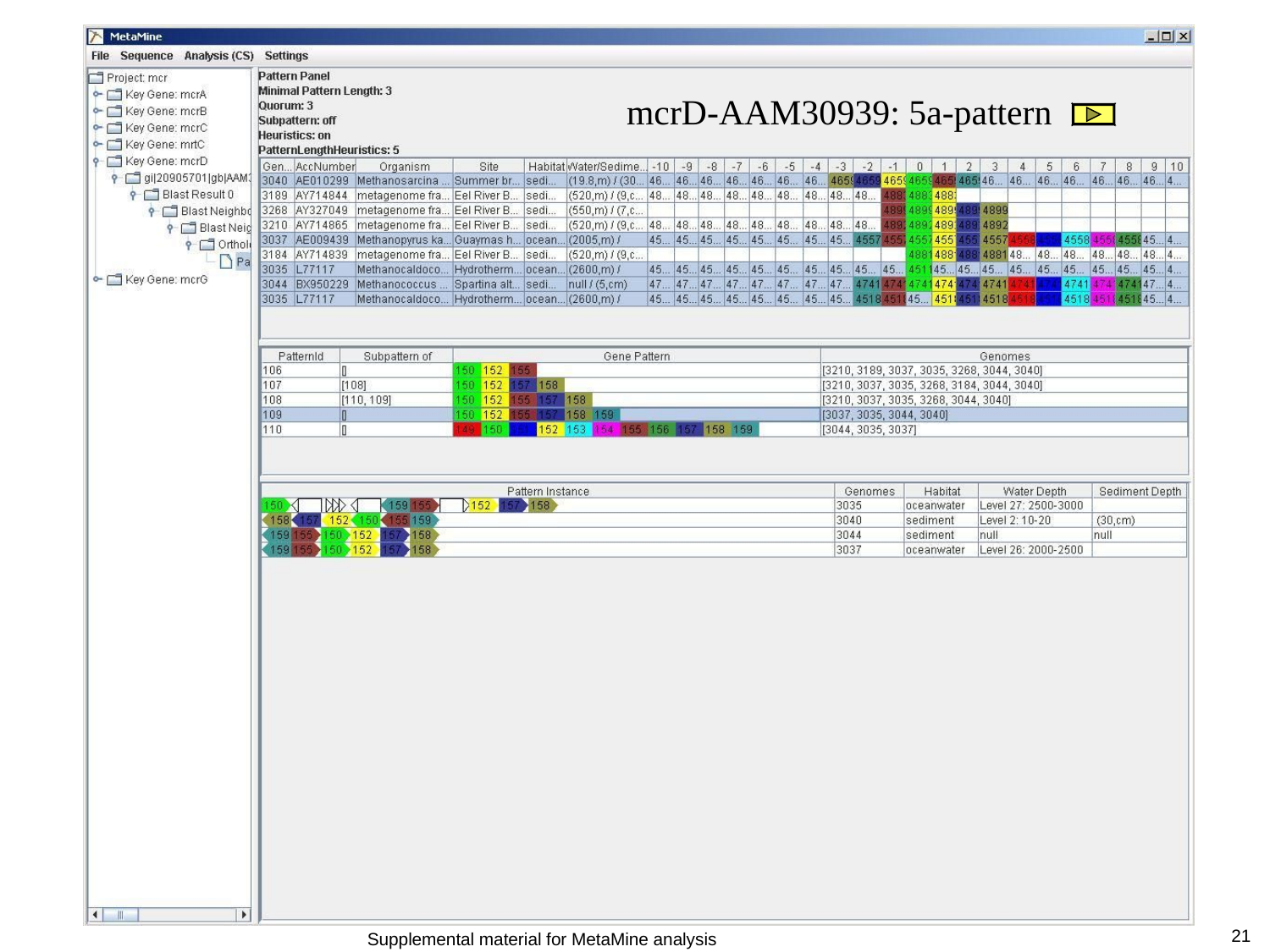

mcrD-AAM30939: 5a-pattern
21

## Slide 22
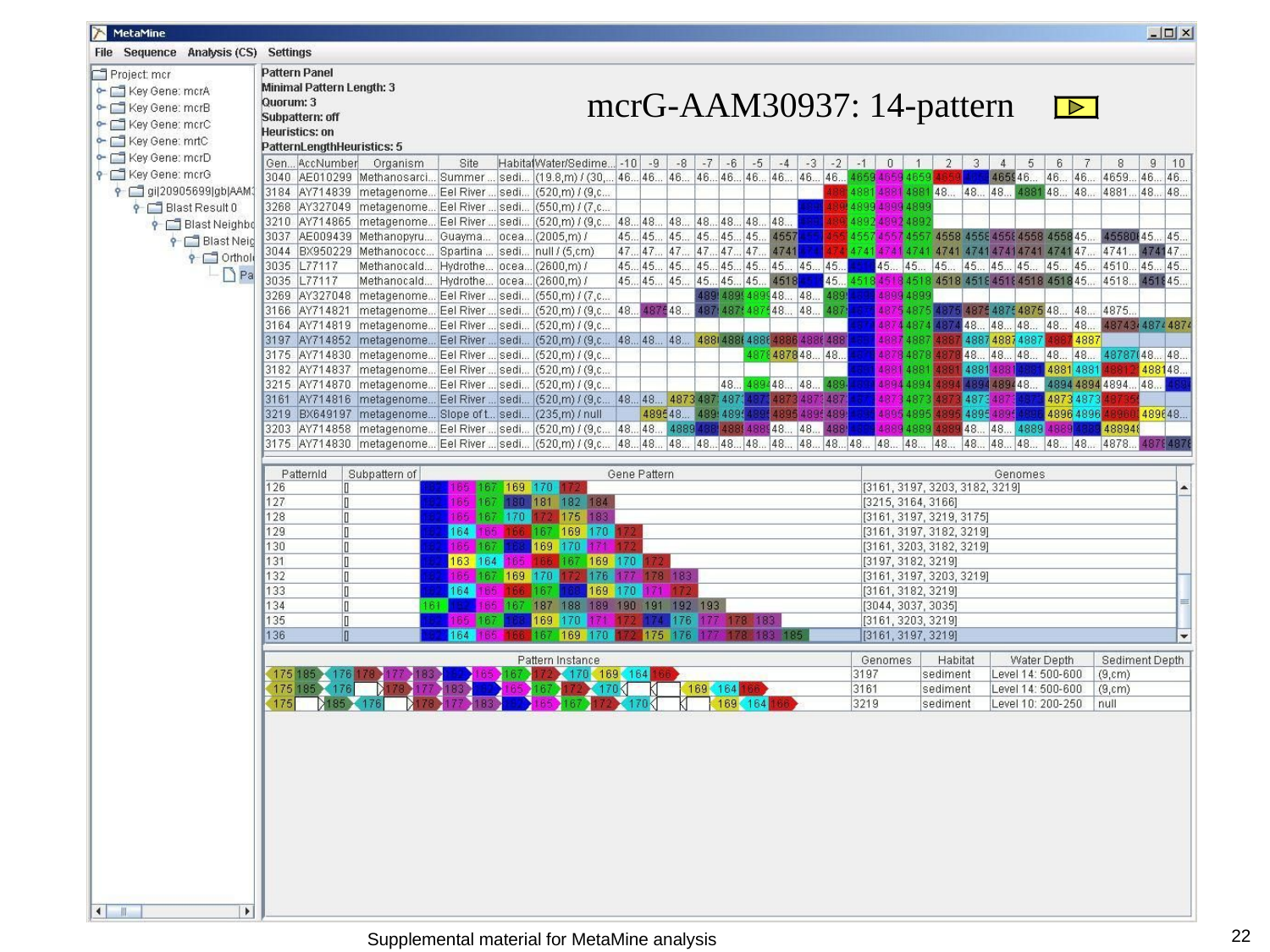

mcrG-AAM30937: 14-pattern
22

## Slide 23
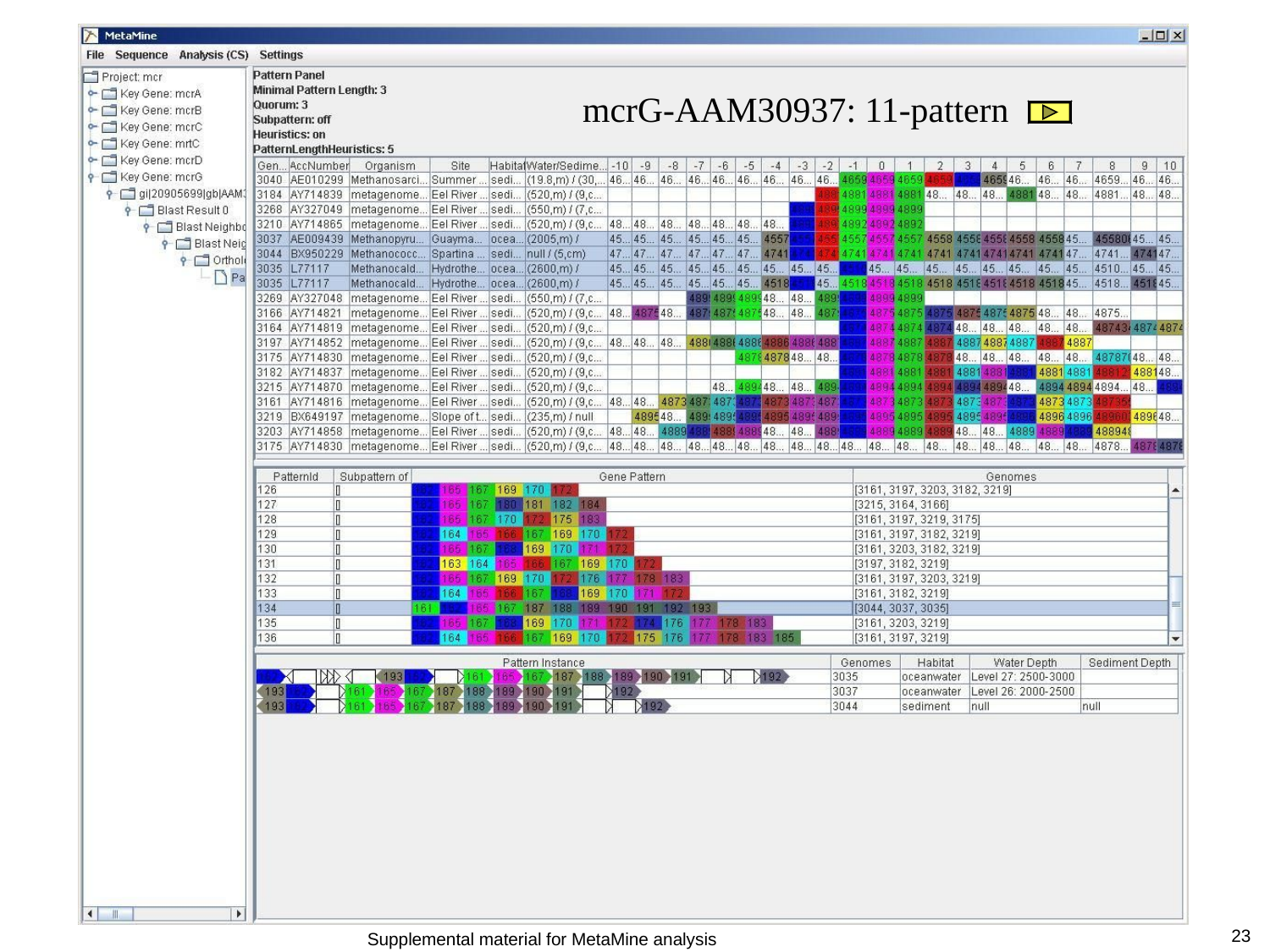

mcrG-AAM30937: 11-pattern
23

## Slide 24
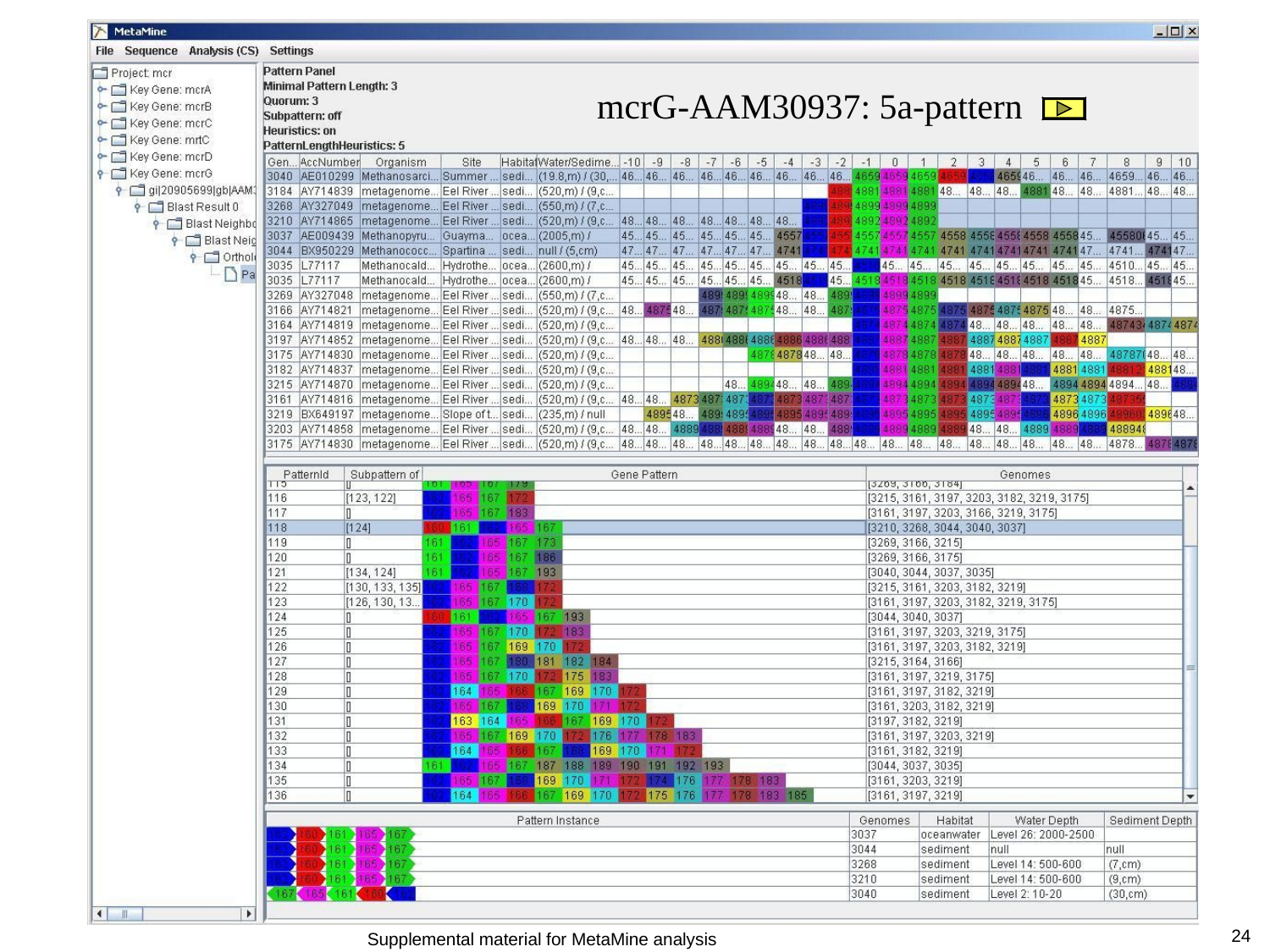

mcrG-AAM30937: 5a-pattern
24

## Slide 25
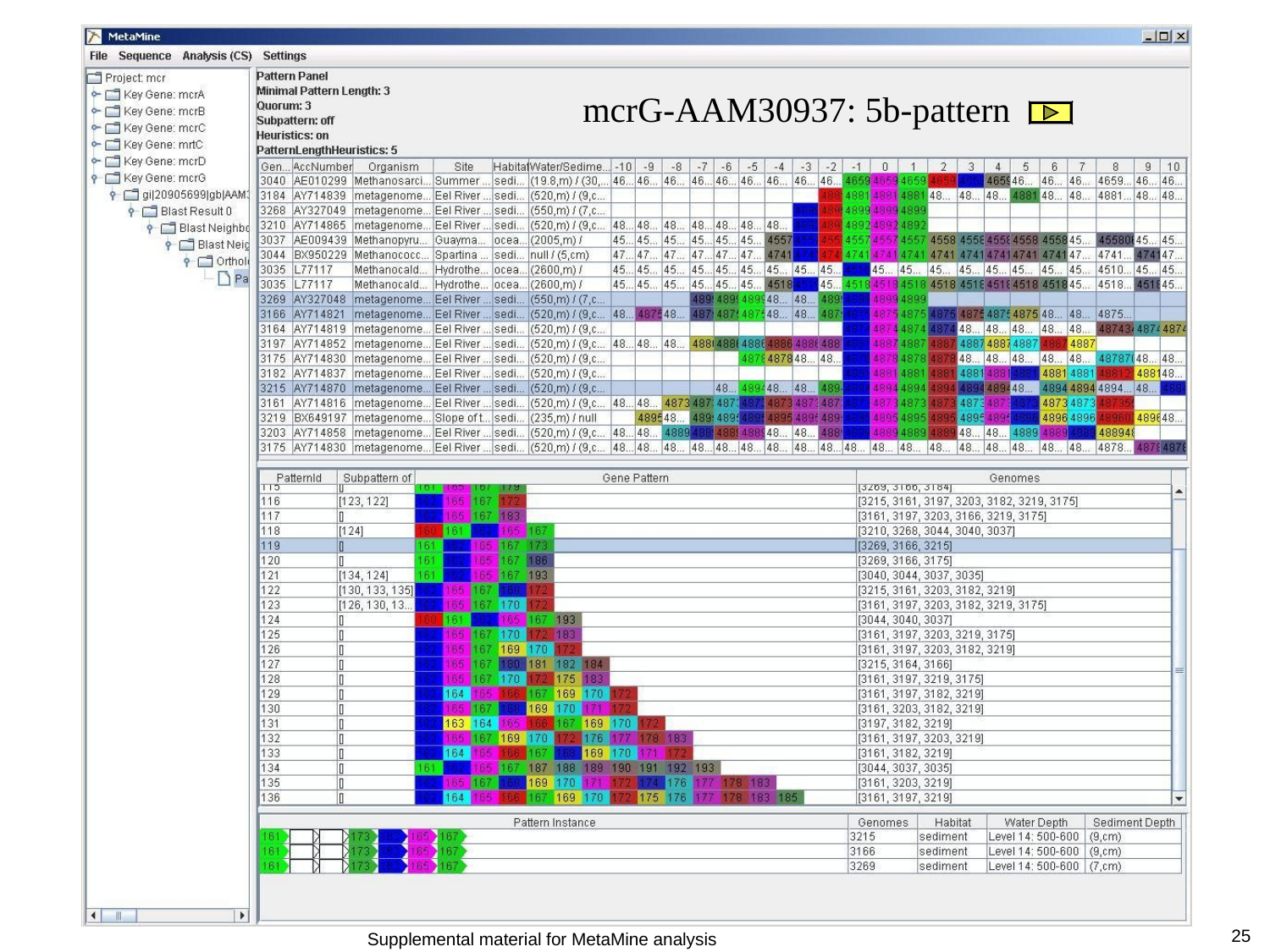

mcrG-AAM30937: 5b-pattern
25

## Slide 26
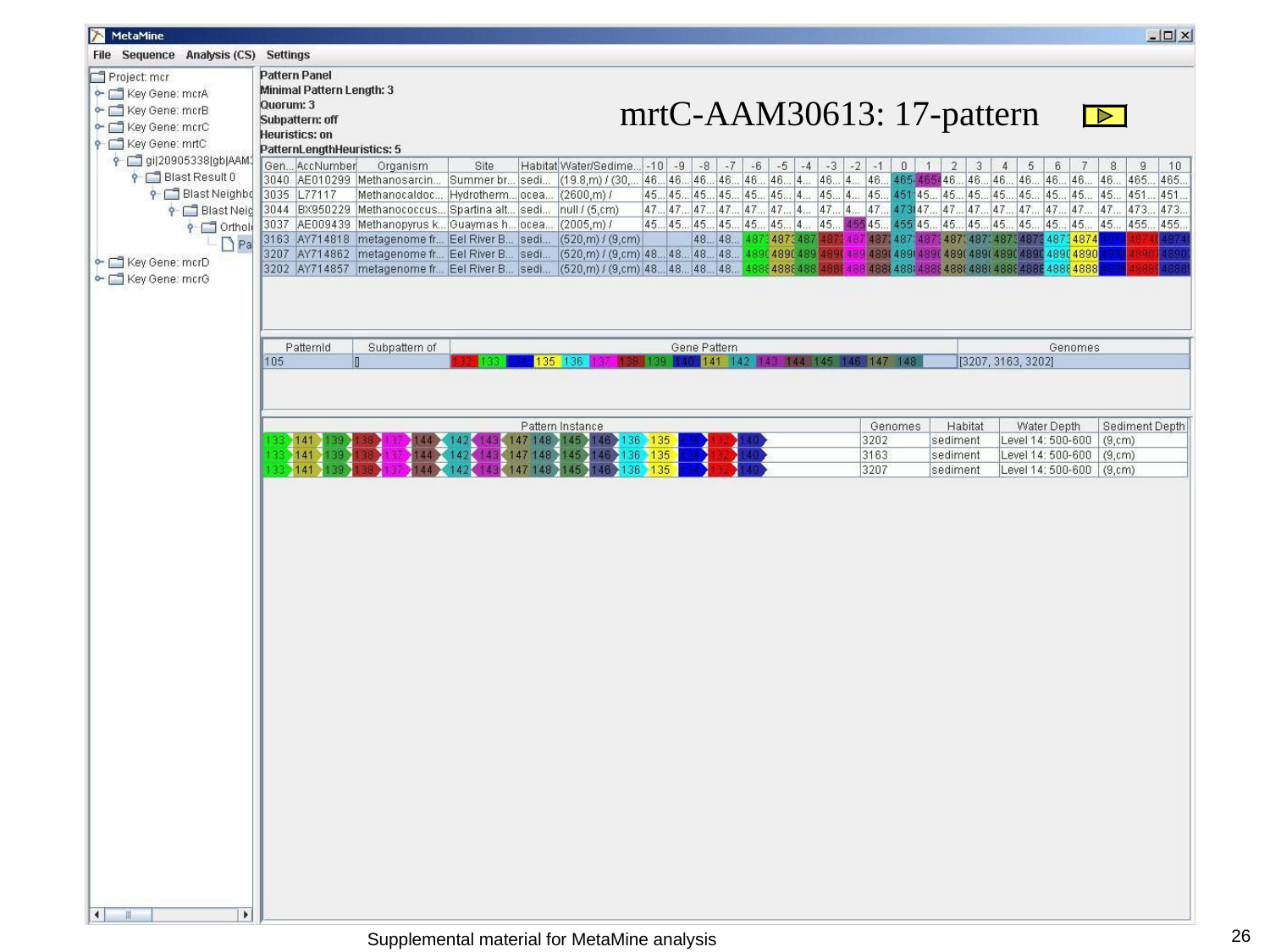

mrtC-AAM30613: 17-pattern
26

## Slide 27
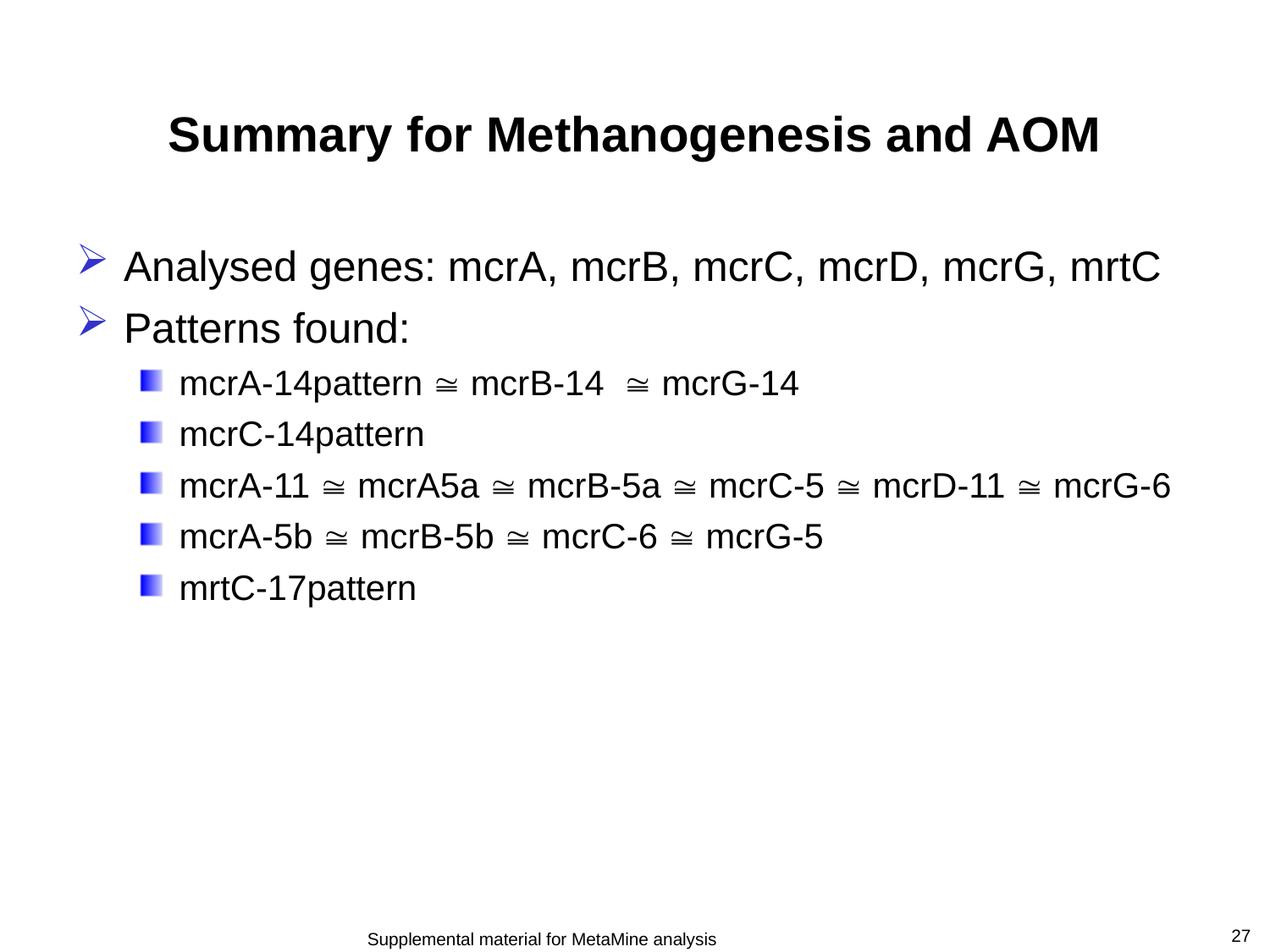

# Summary for Methanogenesis and AOM
Analysed genes: mcrA, mcrB, mcrC, mcrD, mcrG, mrtC
Patterns found:
mcrA-14pattern  mcrB-14  mcrG-14
mcrC-14pattern
mcrA-11  mcrA5a  mcrB-5a  mcrC-5  mcrD-11  mcrG-6
mcrA-5b  mcrB-5b  mcrC-6  mcrG-5
mrtC-17pattern
27

## Slide 28
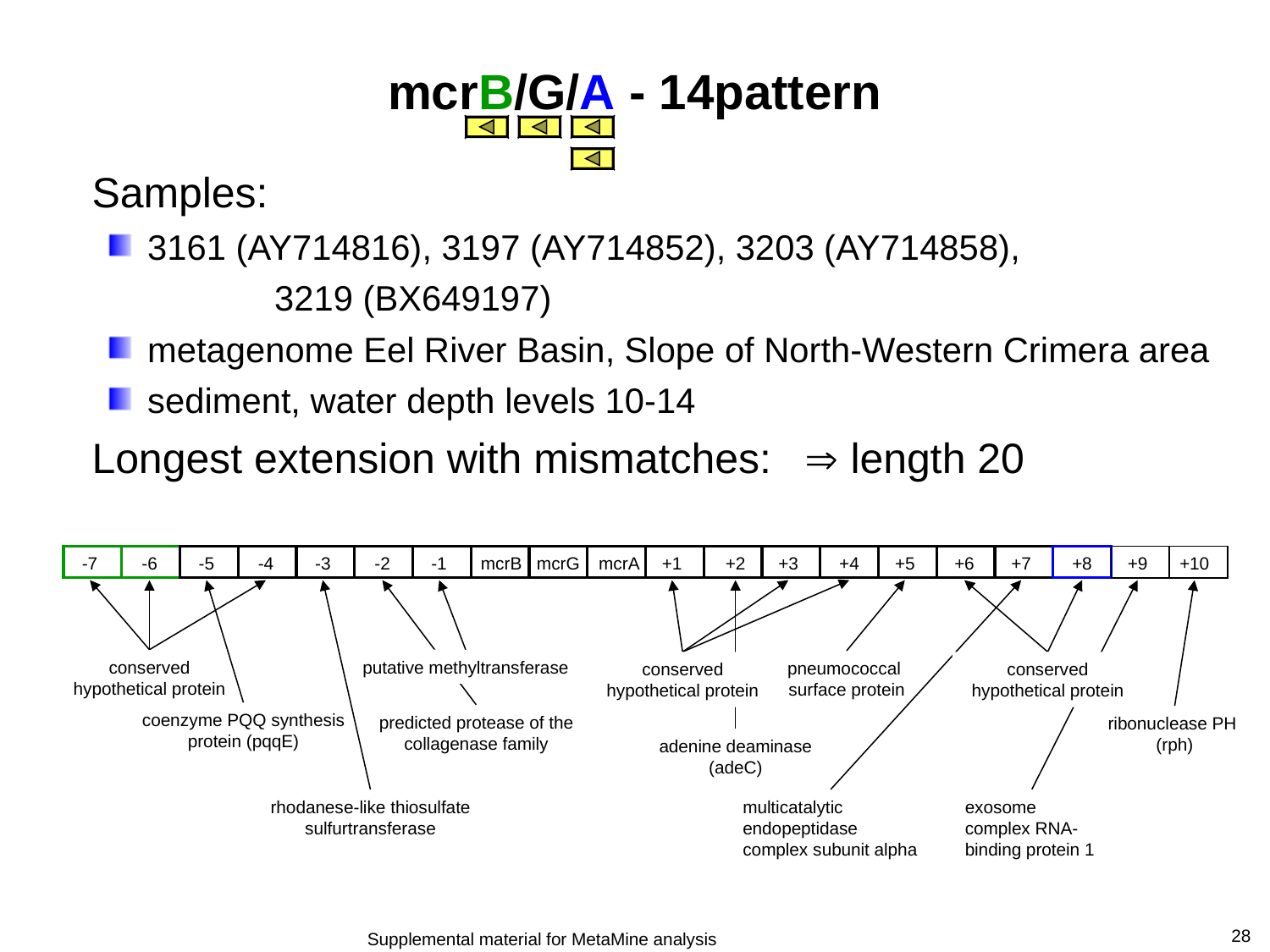

mcrB/G/A - 14pattern
# Samples:
3161 (AY714816), 3197 (AY714852), 3203 (AY714858),
	3219 (BX649197)
metagenome Eel River Basin, Slope of North-Western Crimera area
sediment, water depth levels 10-14
Longest extension with mismatches:  length 20
-7
-6
-5
-4
-3
-2
-1
mcrB
mcrG
mcrA
+1
+2
+3
+4
+5
+6
+7
+8
+9
+10
conserved hypothetical protein
putative methyltransferase
pneumococcal
surface protein
conserved hypothetical protein
conserved hypothetical protein
coenzyme PQQ synthesis protein (pqqE)
predicted protease of the collagenase family
ribonuclease PH
(rph)
adenine deaminase (adeC)
rhodanese-like thiosulfate sulfurtransferase
multicatalytic endopeptidase complex subunit alpha
exosome complex RNA-binding protein 1
28

## Slide 29
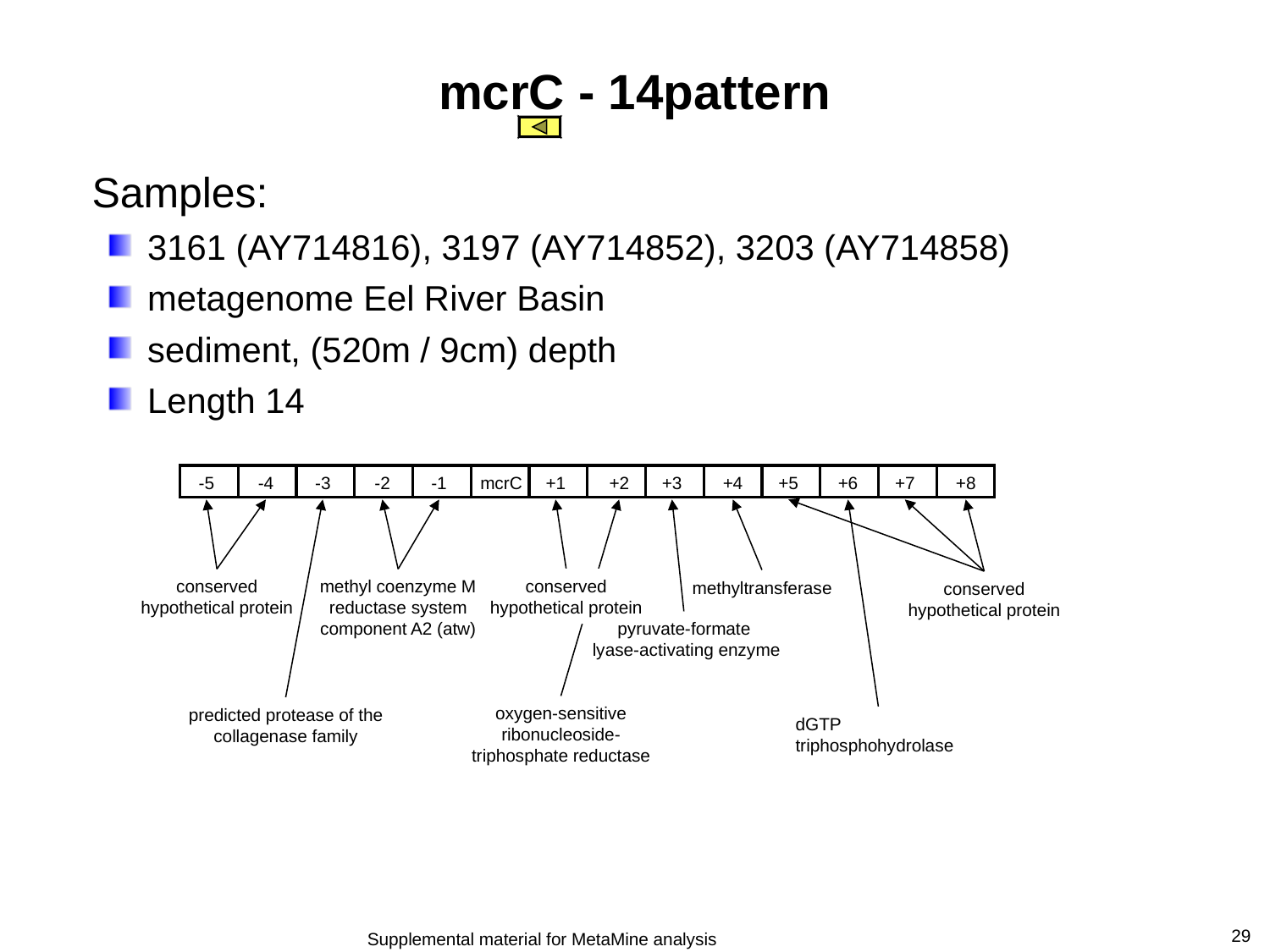

mcrC - 14pattern
# Samples:
3161 (AY714816), 3197 (AY714852), 3203 (AY714858)
metagenome Eel River Basin
sediment, (520m / 9cm) depth
Length 14
-5
-4
-3
-2
-1
mcrC
+1
+2
+3
+4
+5
+6
+7
+8
conserved hypothetical protein
conserved hypothetical protein
methyl coenzyme M reductase system component A2 (atw)
methyltransferase
conserved hypothetical protein
pyruvate-formate
 lyase-activating enzyme
oxygen-sensitive ribonucleoside-triphosphate reductase
predicted protease of the collagenase family
dGTP triphosphohydrolase
29

## Slide 30
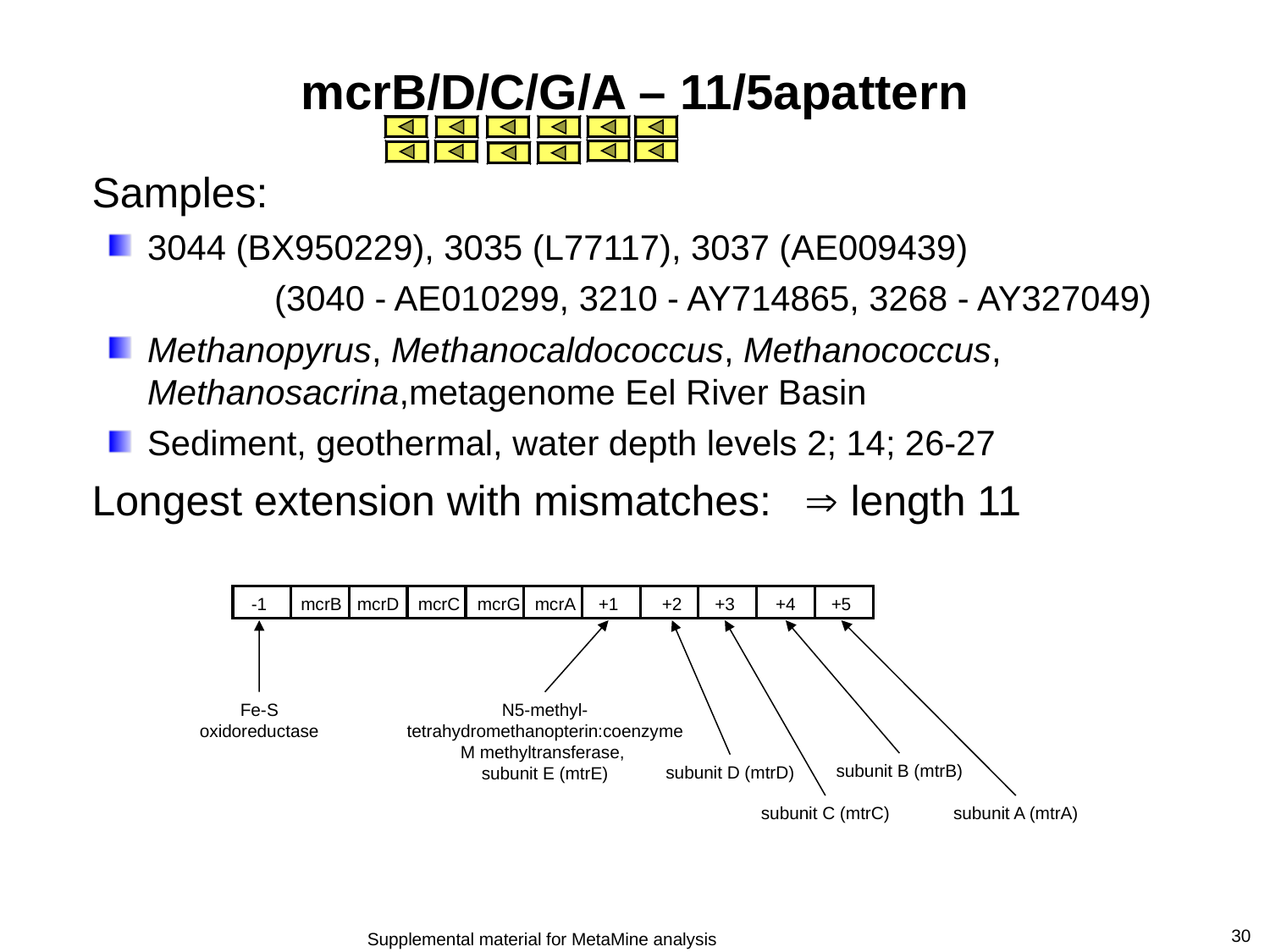

mcrB/D/C/G/A – 11/5apattern
# Samples:
3044 (BX950229), 3035 (L77117), 3037 (AE009439)
	(3040 - AE010299, 3210 - AY714865, 3268 - AY327049)
Methanopyrus, Methanocaldococcus, Methanococcus, Methanosacrina,metagenome Eel River Basin
Sediment, geothermal, water depth levels 2; 14; 26-27
Longest extension with mismatches:  length 11
-1
mcrB
mcrD
mcrC
mcrG
mcrA
+1
+2
+3
+4
+5
Fe-S oxidoreductase
N5-methyl-tetrahydromethanopterin:coenzyme M methyltransferase,
subunit E (mtrE)
subunit B (mtrB)
subunit D (mtrD)
subunit C (mtrC)
subunit A (mtrA)
30

## Slide 31
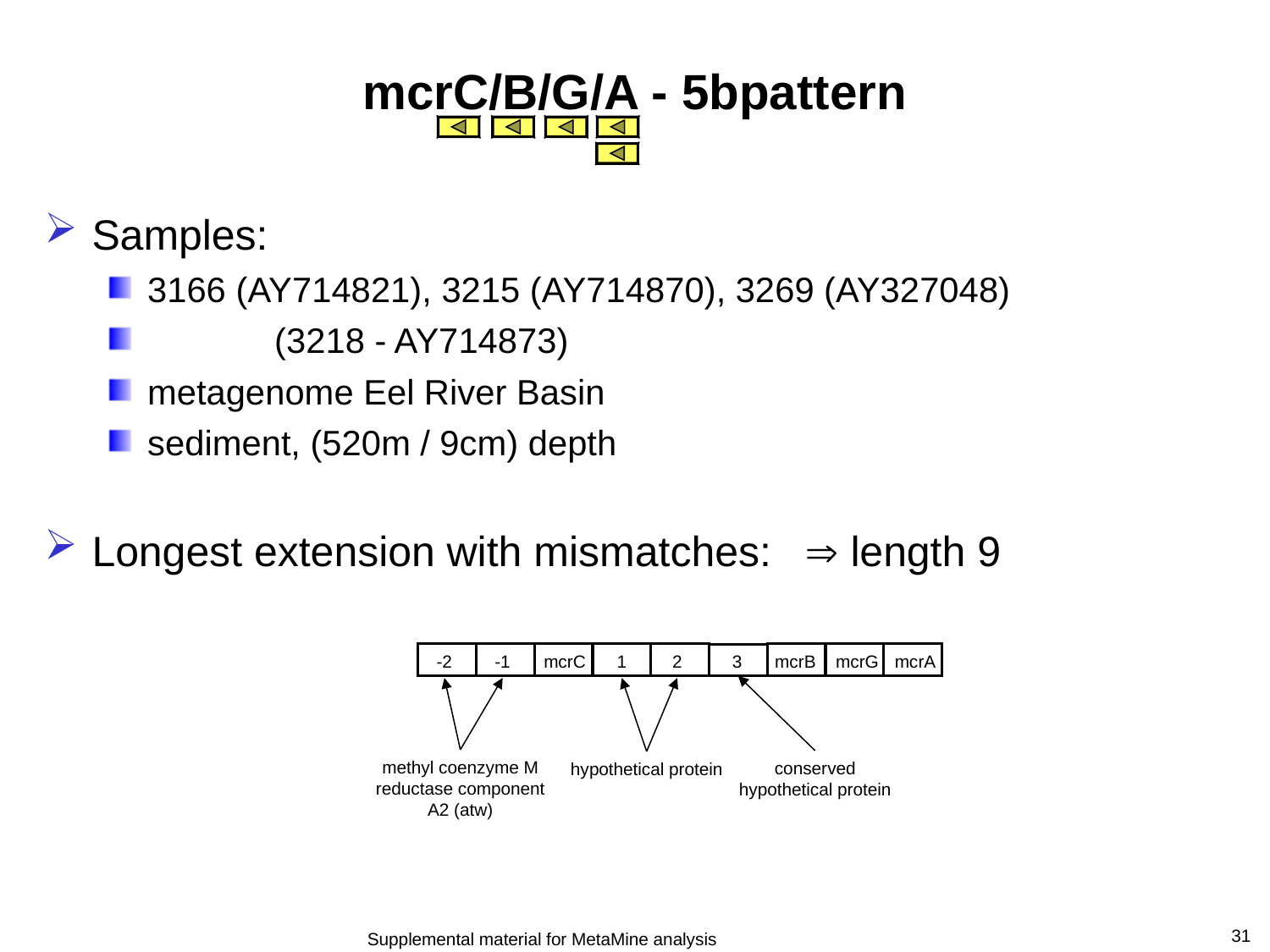

# mcrC/B/G/A - 5bpattern
Samples:
3166 (AY714821), 3215 (AY714870), 3269 (AY327048)
	(3218 - AY714873)
metagenome Eel River Basin
sediment, (520m / 9cm) depth
Longest extension with mismatches:  length 9
-2
-1
mcrC
1
2
mcrB
mcrG
mcrA
3
methyl coenzyme M reductase component A2 (atw)
conserved
hypothetical protein
hypothetical protein
31

## Slide 32
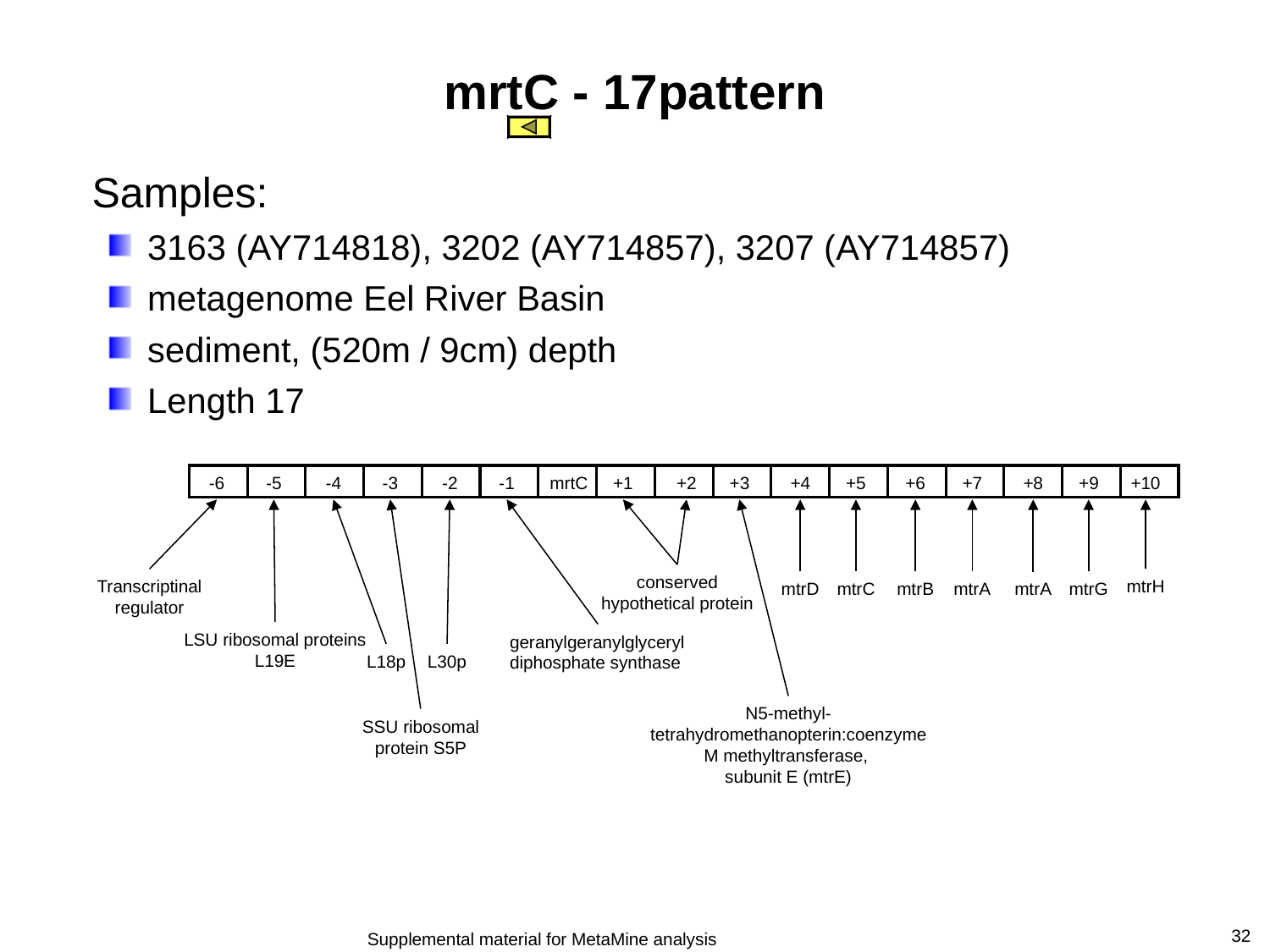

# mrtC - 17pattern
Samples:
3163 (AY714818), 3202 (AY714857), 3207 (AY714857)
metagenome Eel River Basin
sediment, (520m / 9cm) depth
Length 17
-6
-5
-4
-3
-2
-1
mrtC
+1
+2
+3
+4
+5
+6
+7
+8
+9
+10
conserved hypothetical protein
Transcriptinal regulator
mtrH
mtrD
mtrC
mtrB
mtrA
mtrG
mtrA
LSU ribosomal proteins L19E
geranylgeranylglyceryl diphosphate synthase
L18p
L30p
N5-methyl-tetrahydromethanopterin:coenzyme M methyltransferase,
subunit E (mtrE)
SSU ribosomal protein S5P
32

## Slide 33
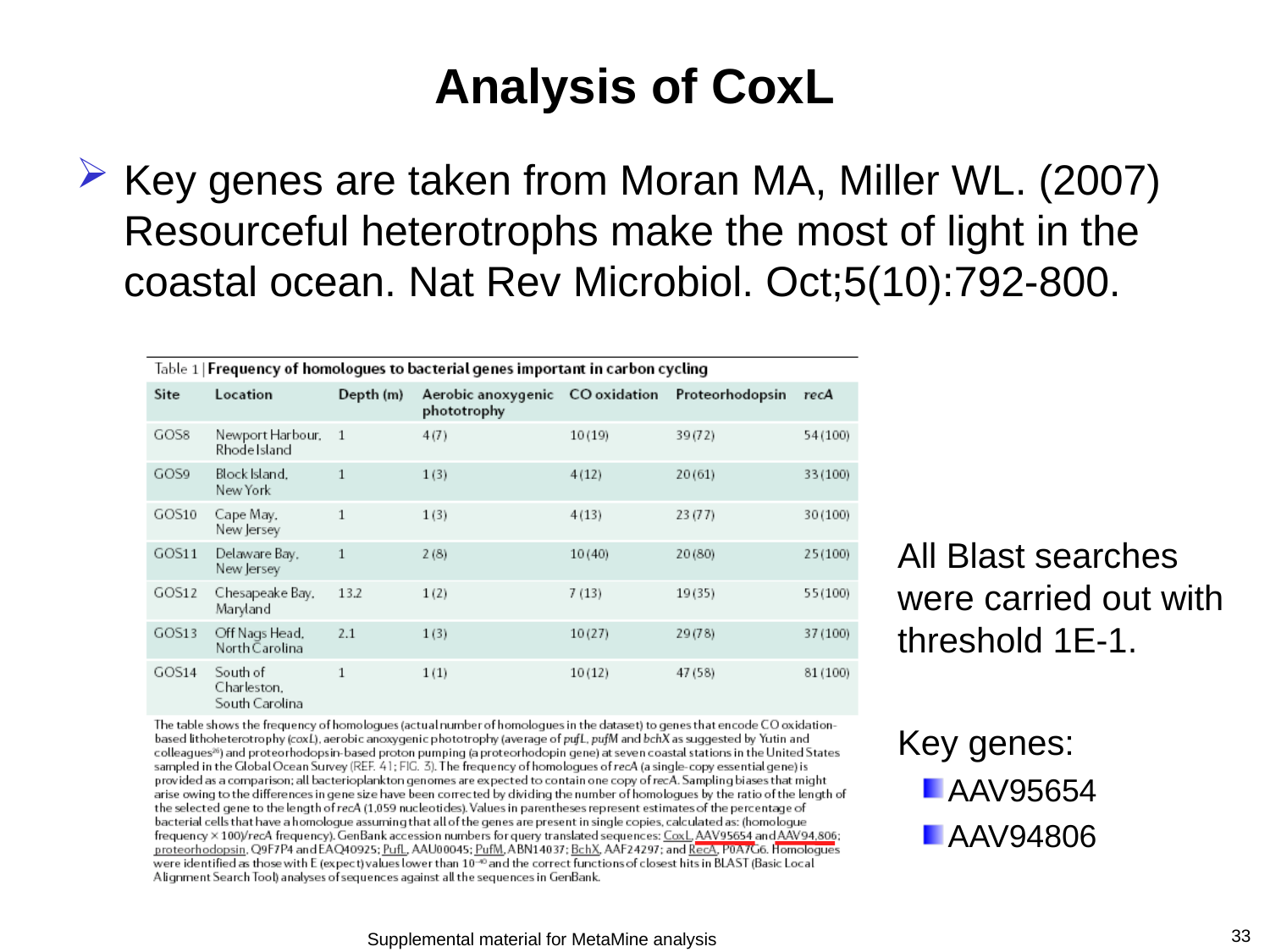

# Analysis of CoxL
Key genes are taken from Moran MA, Miller WL. (2007) Resourceful heterotrophs make the most of light in the coastal ocean. Nat Rev Microbiol. Oct;5(10):792-800.
All Blast searches were carried out with threshold 1E-1.
Key genes:
AAV95654
AAV94806
33

## Slide 34
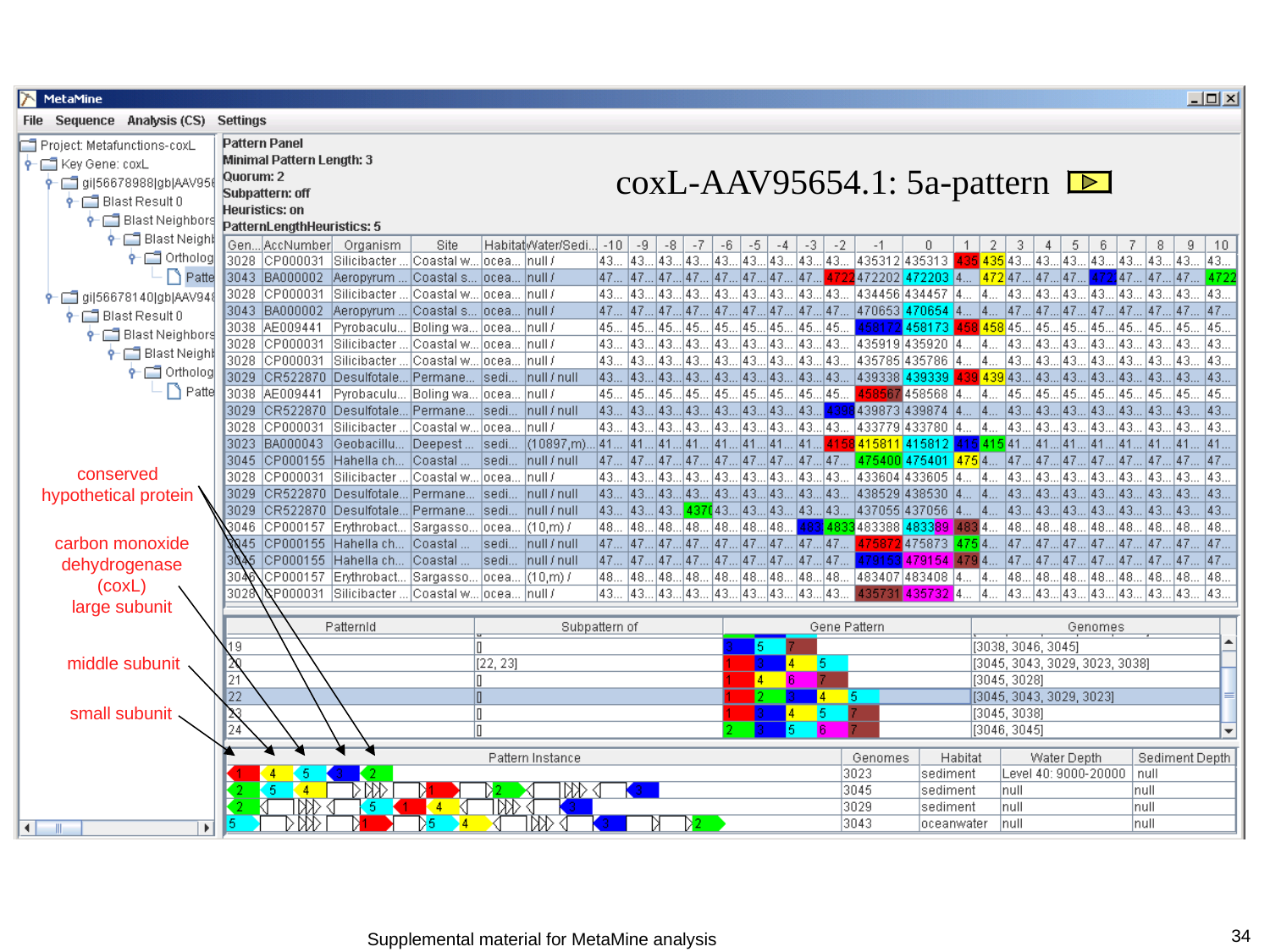

coxL-AAV95654.1: 5a-pattern
conserved hypothetical protein
carbon monoxide dehydrogenase (coxL)
large subunit
middle subunit
small subunit
34

## Slide 35
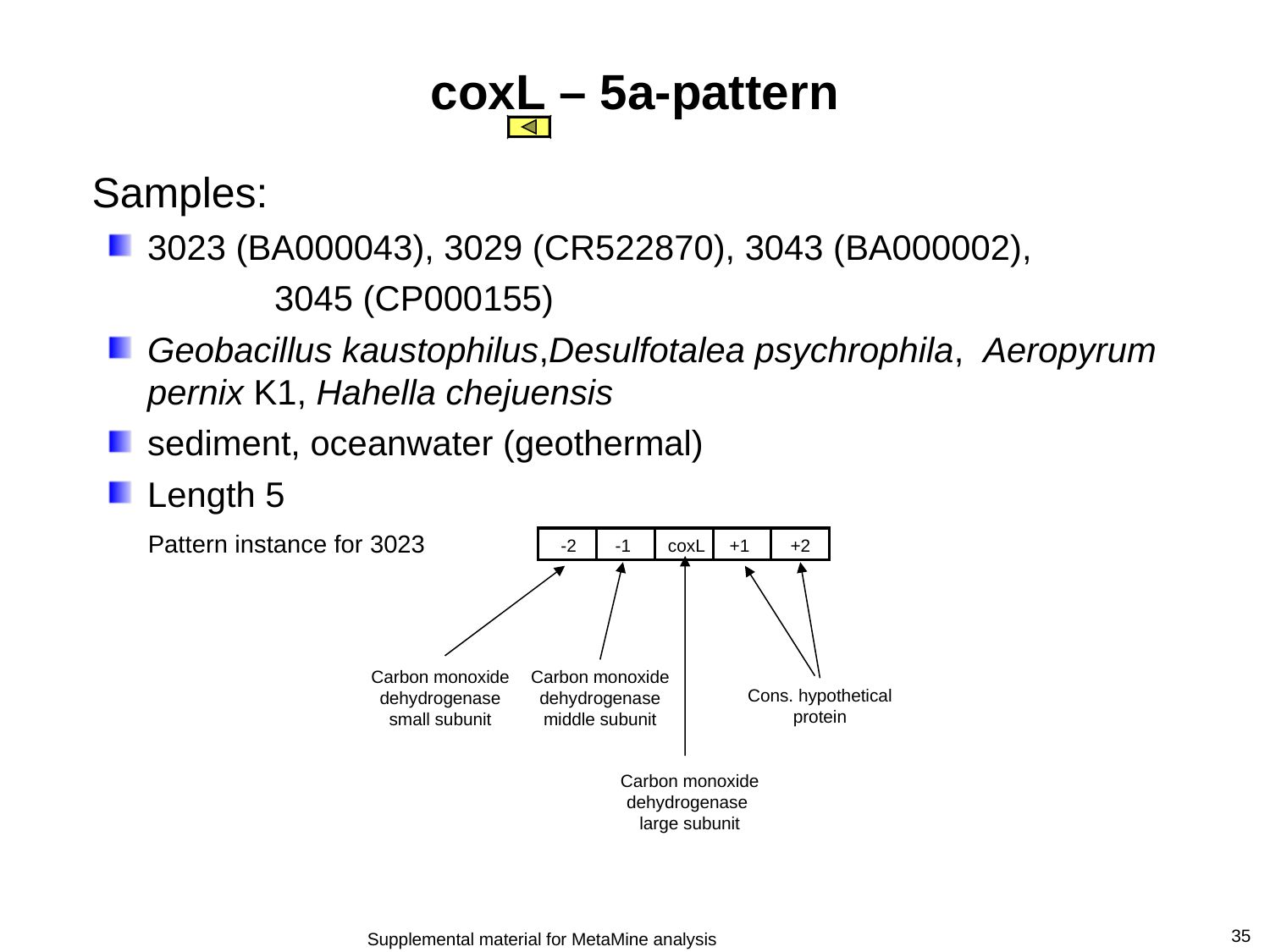

# coxL – 5a-pattern
Samples:
3023 (BA000043), 3029 (CR522870), 3043 (BA000002),
	3045 (CP000155)
Geobacillus kaustophilus,Desulfotalea psychrophila, Aeropyrum pernix K1, Hahella chejuensis
sediment, oceanwater (geothermal)
Length 5
Pattern instance for 3023
-2
-1
coxL
+1
+2
Carbon monoxide dehydrogenase small subunit
Carbon monoxide dehydrogenase middle subunit
Cons. hypothetical protein
Carbon monoxide dehydrogenase
large subunit
35

## Slide 36
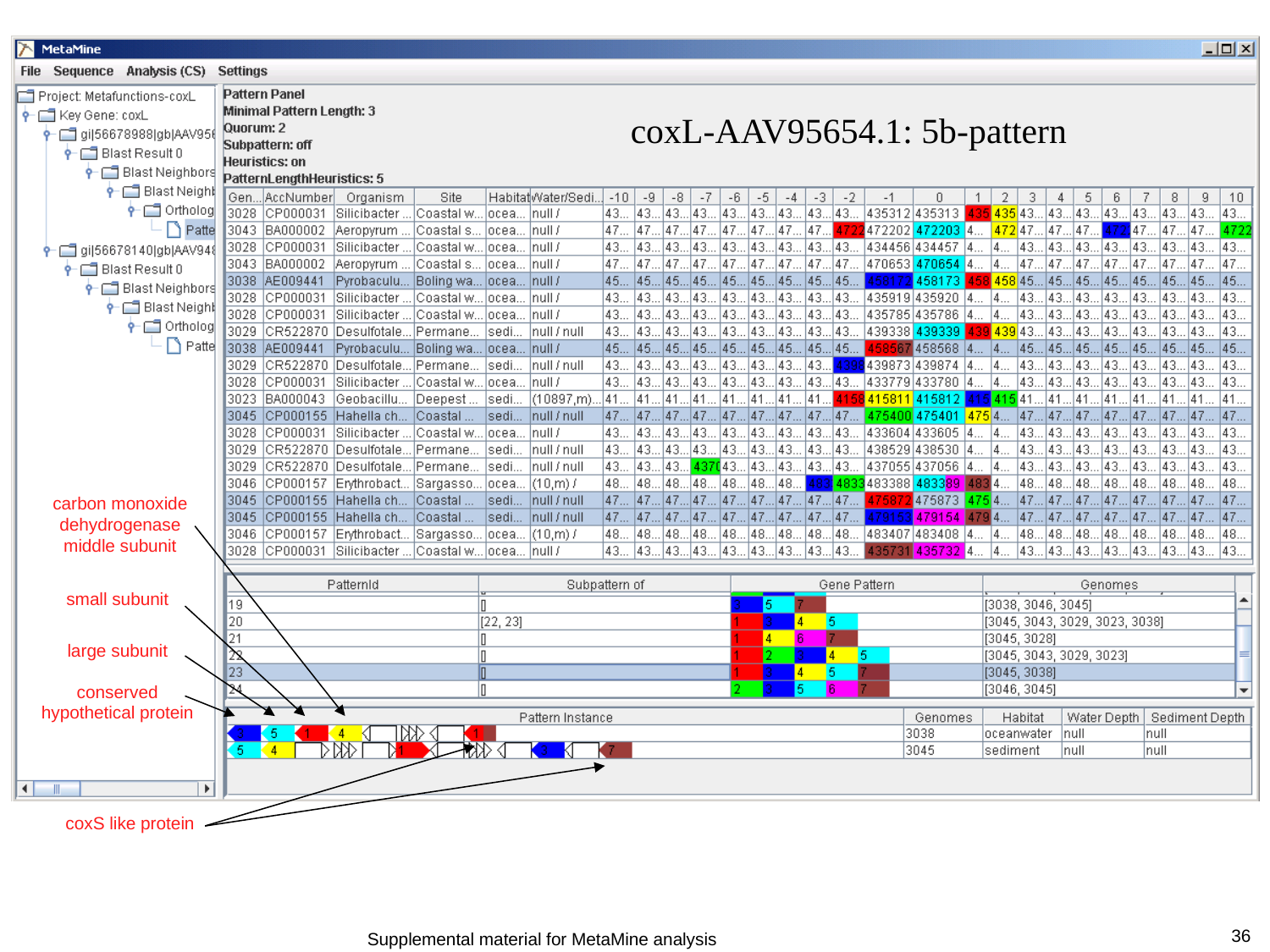

coxL-AAV95654.1: 5b-pattern
carbon monoxide dehydrogenase
middle subunit
small subunit
large subunit
conserved hypothetical protein
coxS like protein
36

## Slide 37
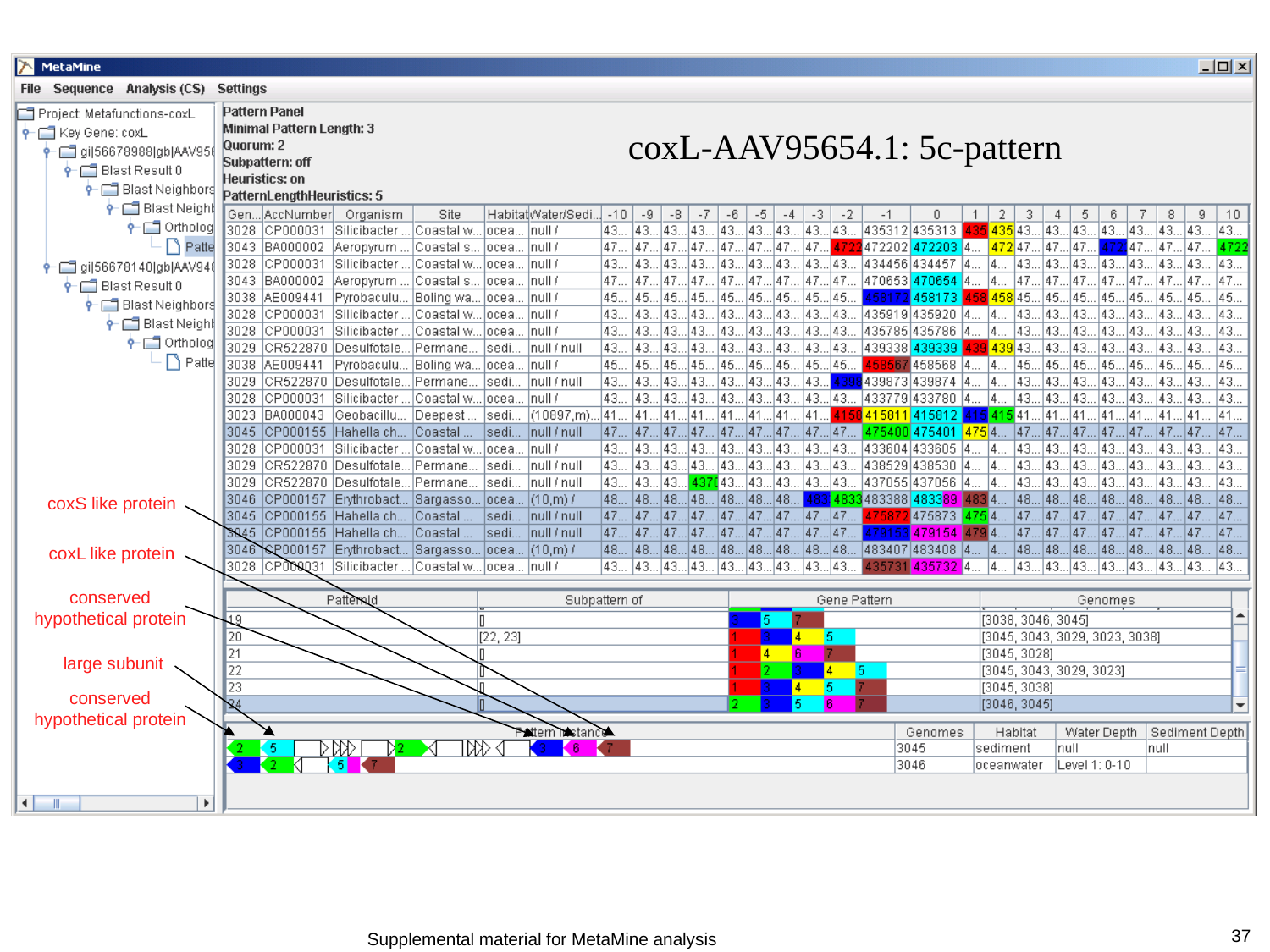

coxL-AAV95654.1: 5c-pattern
coxS like protein
coxL like protein
conserved hypothetical protein
large subunit
conserved hypothetical protein
37

## Slide 38
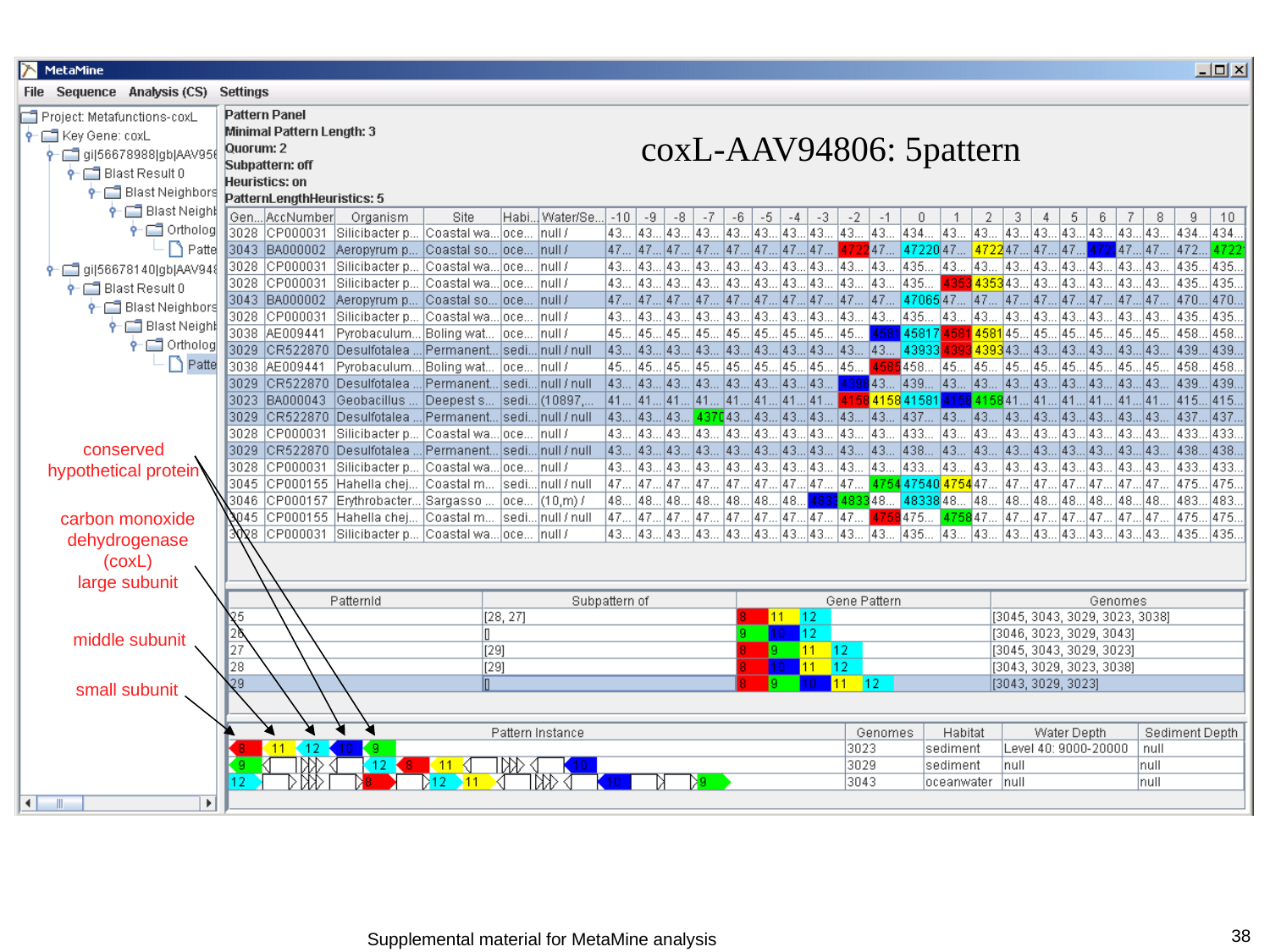

coxL-AAV94806: 5pattern
conserved hypothetical protein
carbon monoxide dehydrogenase (coxL)
large subunit
middle subunit
small subunit
38
