# Supplementary material for: MetaMine – A tool to detect and analyse gene patterns in their environmental context
Source: BMC Bioinformatics. 2008 Oct 28;9:459. doi: 10.1186/1471-2105-9-459 (PMC2615450; doi:10.1186/1471-2105-9-459)
Supplement: Additional file 2 — The file contains a user guide about MetaMine version 1.2. [file 1471-2105-9-459-S2.doc]

**MetaMine 1.2 Documentation**

<http://www.megx.net/metamine/>

September, 2008

Uta Bohnebeck, Renzo Kottmann, Thierry Lombardot, Frank Oliver Glöckner

Contact address in case of bugs, problems, questions, improvements:

bohnebeck@ttz-bremerhaven or megx@mpi-bremen.de

MetaMine is an interactive data mining tool which enables the detection of gene patterns in an environmental context. The underlying search strategy follows a bottom-up approach starting with a *key gene* which is selected by the user based on prior biological knowledge about its environmental relevance.

The standard search process consists of the following steps:

| 1. Definition of a project 2. Definition of a key gene 3. Import of the corresponding protein sequence 4. BLAST search with the imported key gene sequence 5. Determination of neighboring genes 6. BLAST search of all neighboring genes 7. Determination of functionally equivalent genes 8. Determination of gene patterns 9. Save/Export the project results | Parameters:  - Min. pattern length  - Quorum  - Subpatterns  - Heuristics  - Pattern lengths heuristics  Parameters:  - number of neighbors  - overlap buffer  Blast Neighbors Result  Vector of Neighbors  Project  Key Gene  Gene Sequence  Definition of project  Definition of key gene  Determination of gene patterns  List of Gene Patterns  Blast Result  Parameters:  - matrix  - e-value  BLAST search with key gene  Import of protein sequence  Parameters:  - matrix  - e-value  BLAST search of neighbors  List of Functional Groups  Determination of neighbors  Determination of functionally equivalent genes |
| --- | --- |

Each process step can be repeated with other parameters resulting in a tree structure to organise and visualize intermediate results. As shown in the left panel of Fig. 1, the user can navigate through the history of all steps to analyse the corresponding results in the right data panel in more detail.

Note, in order to proceed with the analysis first select the data object in the navigation tree in the control panel and then choose the corresponding action from the menu bar.

Figure 1


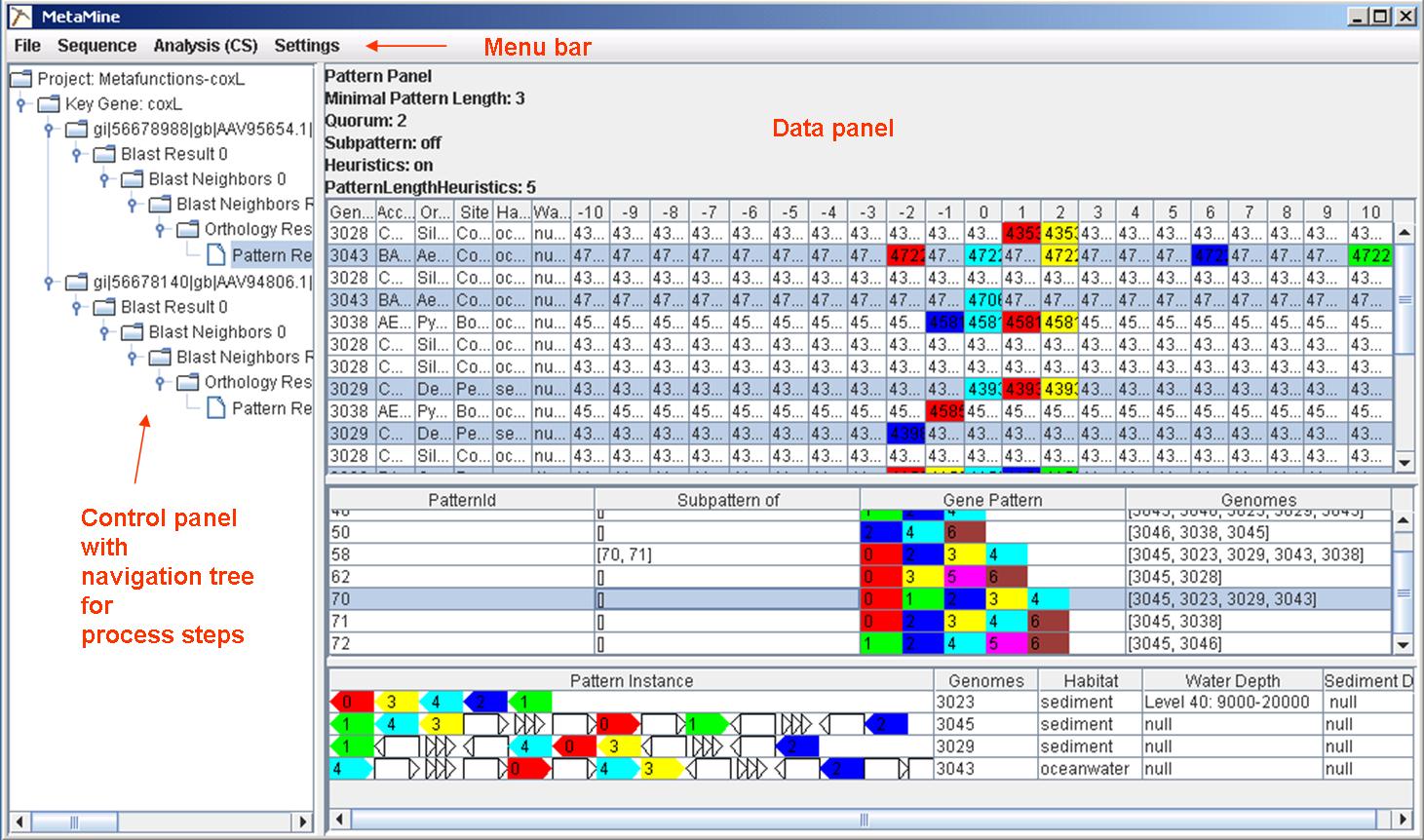


**1. Definition of a project**

After you have started the MetaMine client the first step is to create a project (see Fig. 2). Choose item “New Project” from the File menu to enter the project name, the user name, and a short description of your project.

Press the accept button in order to store this information. A project identifier is generated for internal use.

Figure 2


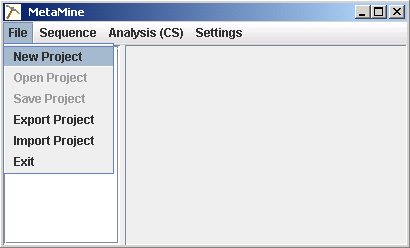


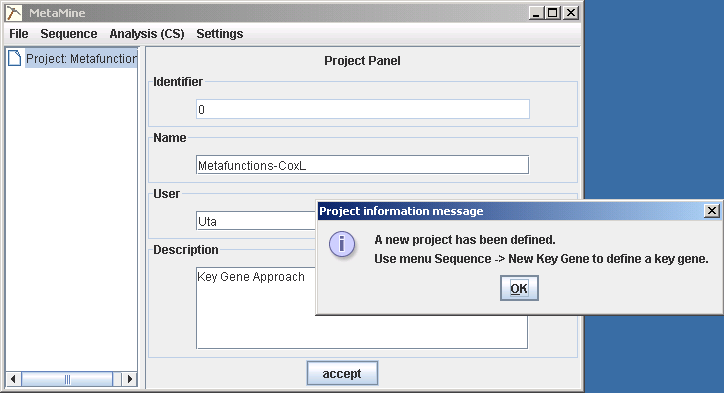


Next step: Definition of a key gene

**2. Definition of a key gene**

Choose item “New Key Gene” from the Sequence menu. Define your key gene by name, function and description and press the accept button to store the information (see Fig. 3).

Figure 3


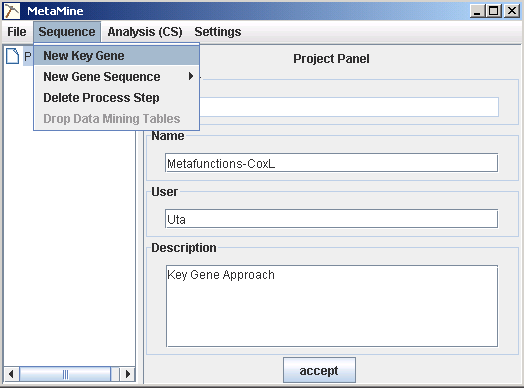


Next step: Import of the corresponding protein sequence

**3. Import of a protein sequence**

After defining the key gene in the control panel choose item “Import Sequence from Fasta File” from the Sequence menu and navigate to the location of your Fasta file using the file dialog, see Fig 4.

After successful upload, the sequence and the corresponding information is shown in the Sequence panel, see Fig. 5

Figure 4


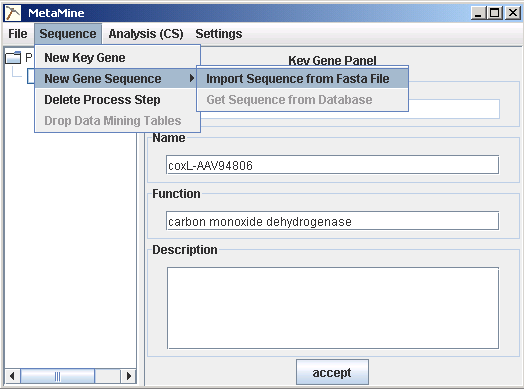


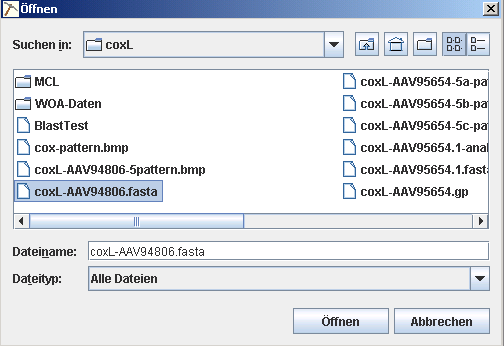


Figure 5


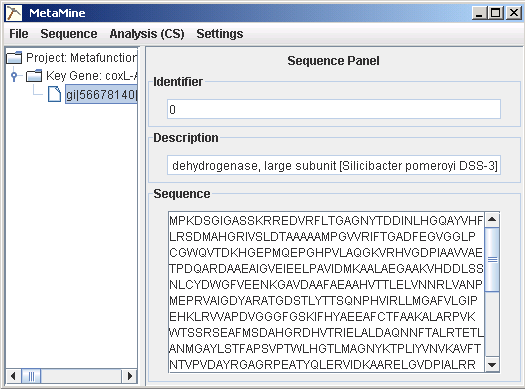


Next step: BLAST search

**General parameter settings:**

Each process step from the Analysis menu is associated with default parameters which can be changed by choosing menu item “Set Parameters” from the Settings menu, see Fig 6. “Mouse Over” provides additional information for each parameter.

Figure 6


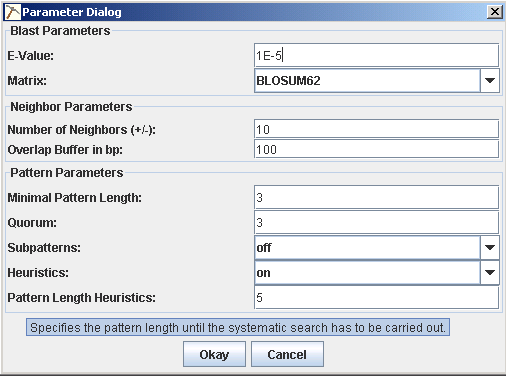


**4. Run the BLAST search:**

Given the protein sequence in the control panel choose menu item “Blast Key Gene” from the Analysis menu, see Figure 7.

Default parameters:

- E-value (used as upper boundary): 1E-5
- Similarity matrix: BLOSUM62

Figure 7


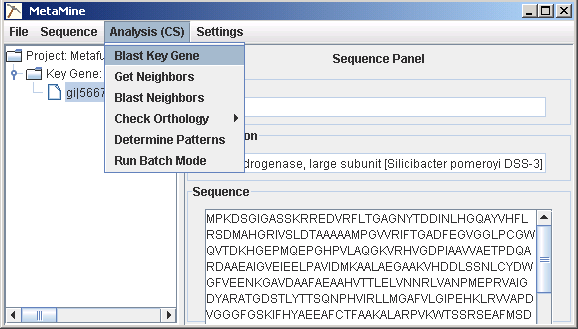


The result of the BLAST search is presented in a table showing for instance e-values, genome information, functional descriptions and schematic alignments (Fig. 8). Click on tab “Meta Information” to see the parameter setting used for this BLAST search.

Figure 8


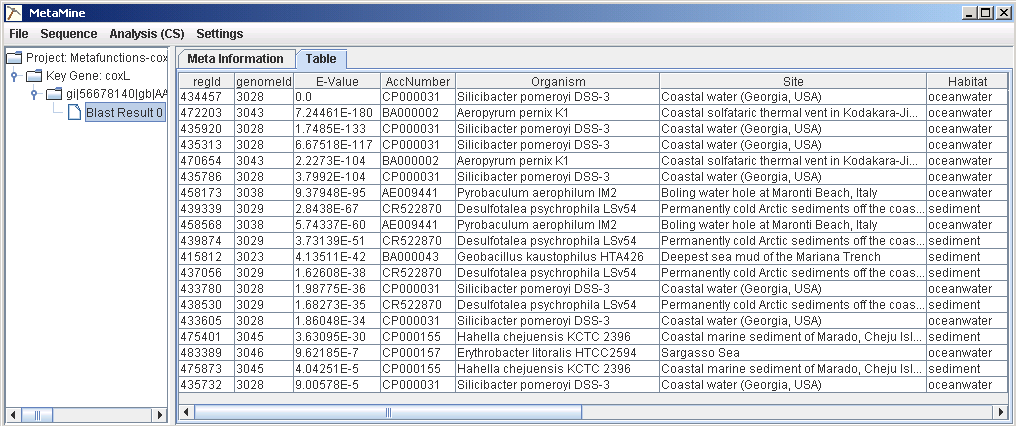


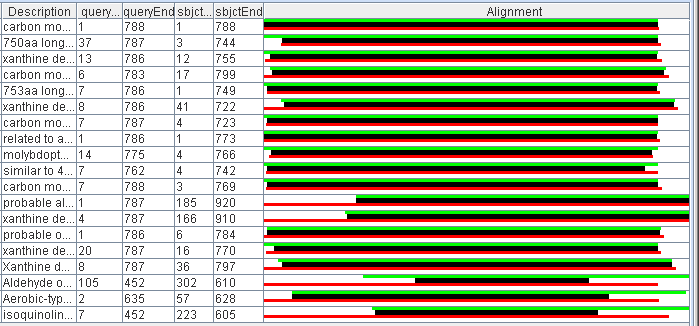


Next step: Determine neighboring genes

**5. Determination of neighboring genes:**

Given the BLAST result choose menu item “Get Neighbors” from the Analysis menu.

Default parameters (Fig. 6):

- Number of neighbors to each side: 10

Maximum number of neighboring genes allowed up and downstream of the key gene

- Overlap Buffer in bp: 100

Specifies the number of base pairs two adjacent genes are allowed to overlap.

The Neighbor panel (Fig. 9) is divided into two parts. The upper part shows a table with all neighboring genes. By clicking on a gene, detailed annotation information is shown in the lower panel including accession number, start and stop position within the genome sequence and the functional description.

Figure 9


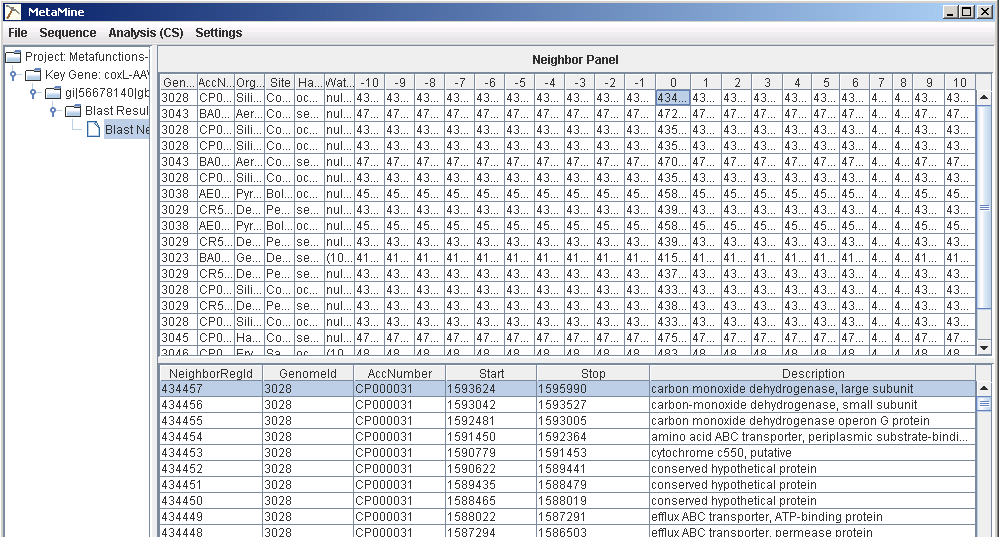


Next step: BLAST search of all neighboring genes

**6. BLAST search of all neighboring genes**

After calculating the neighboring genes choose menu item “Blast Neighbors” from the Analysis menu. While the BLAST process is running a progress bar is shown.

Default parameters (Fig 6):

- E-value (used as upper boundary): 1E-5
- Similarity matrix: BLOSUM62

A mouse over action shows the annotation of a gene. By clicking on a gene the corresponding BLAST result is presented in the lower part of the data panel (see also Fig. 8).

Figure 10


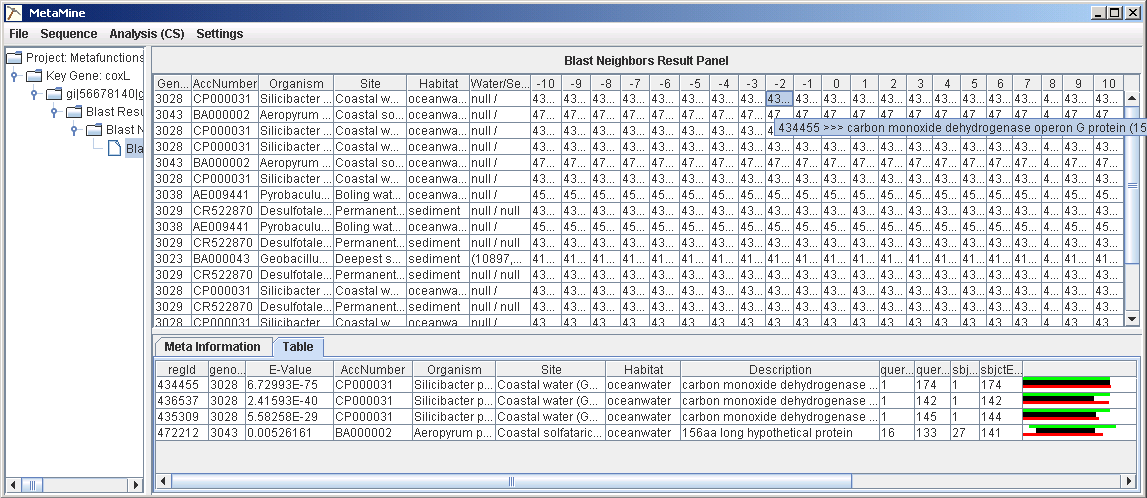


Next step: Determination of functionally equivalent genes

**7. Determination of functionally equivalent genes**

In the current version of MetaMine a COG-based approach (Tatusov *et al*., 2000) to determine functionally equivalent genes is implemented. This procedure consists of three steps:

- For each gene from the upper table: Determine the best match to each (meta)genome
- Filter these matches to be reciprocal best matches
- Create groups of functionally equivalent genes based on triangle relationships


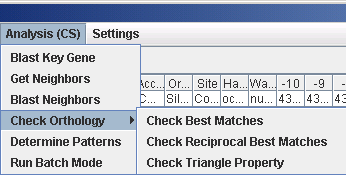


These three steps correspond to the menu items

- “Check Best Matches”,
- “Check Reciprocal Best Matches”
- “Check Triangle Property”

in the Analysis menu.

The Orthology panel (Fig. 11) is divided into three parts:

1. the upper table with the neighboring genes,
2. the middle table showing the best and reciprocal best matches depending with analysis step was chosen and
3. the lower table showing the functional groups together with their colour-coding and their associated genes.

Again, the annotation of a gene can be seen by a mouse over action. By clicking on a gene the corresponding (reciprocal) best matches and the functional group (cluster) for this gene are highlighted (see Fig. 11).

Figure 11


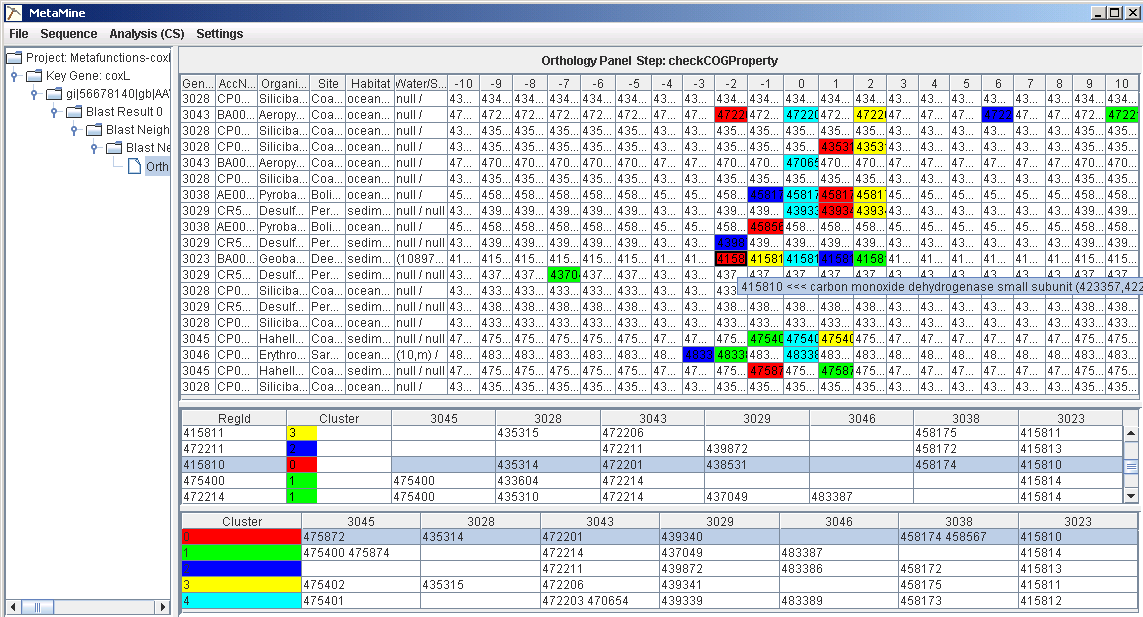


Next step: Determine gene patterns

**8. Determination of gene patterns**

Given the functional groups choose item “Determine Patterns” from the Analysis menu. While this process is running a progress bar is shown.

Default parameters (Fig. 6):

- Minimal pattern length: 3

Specifies the minimal number of genes the pattern must contain.

- Quorum: 3

Specifies the minimal number of (meta)genome sequences where the pattern occurs.

- Subpatterns: off

Specifies whether all subpatterns should be shown.

- Heuristics: on

Specifies whether a heuristic should be used instead of a systematic search.

- Pattern Length Heuristics: 5

Specifies the pattern length until the systematic search has to be carried out.

Using the default parameters the user will obtain a maximal amount of redundance-free gene patterns, excluding patters below a length of three genes. To focus on more frequent patterns the user can increase the parameter *quorum*. Increasing the parameter *minimal pattern length* results in a lower number of sequences where a pattern is present. Higher values for any of the two parameters speed up the search process.

If the parameter *subpatterns* is activated, all subpatterns will be shown, but be careful, this can be a huge number. If this parameter is deactivated, only subpatterns covering more sequences than the corresponding longer pattern will be shown. If this is the case even relatively short patterns, which are not subpatterns of any longer pattern, may be of special interest because they might point to genes with an unusual occurrence.

The Pattern panel (Fig. 12) is divided into three parts:

1. the upper table with the neighboring genes,
2. the middle table showing all patterns found and
3. the lower table with the corresponding pattern instances.

The annotation of a gene can be seen by a mouse over action. By clicking on a gene/ genome sequence all patterns are highlighted where this sequence is associated with. By clicking on a pattern all corresponding pattern instances (describing gene order and directions) are shown together with the environmental parameters of the sample. In addition, the corresponding genome sequences are marked in the table with the neighboring genes.

Figure 12


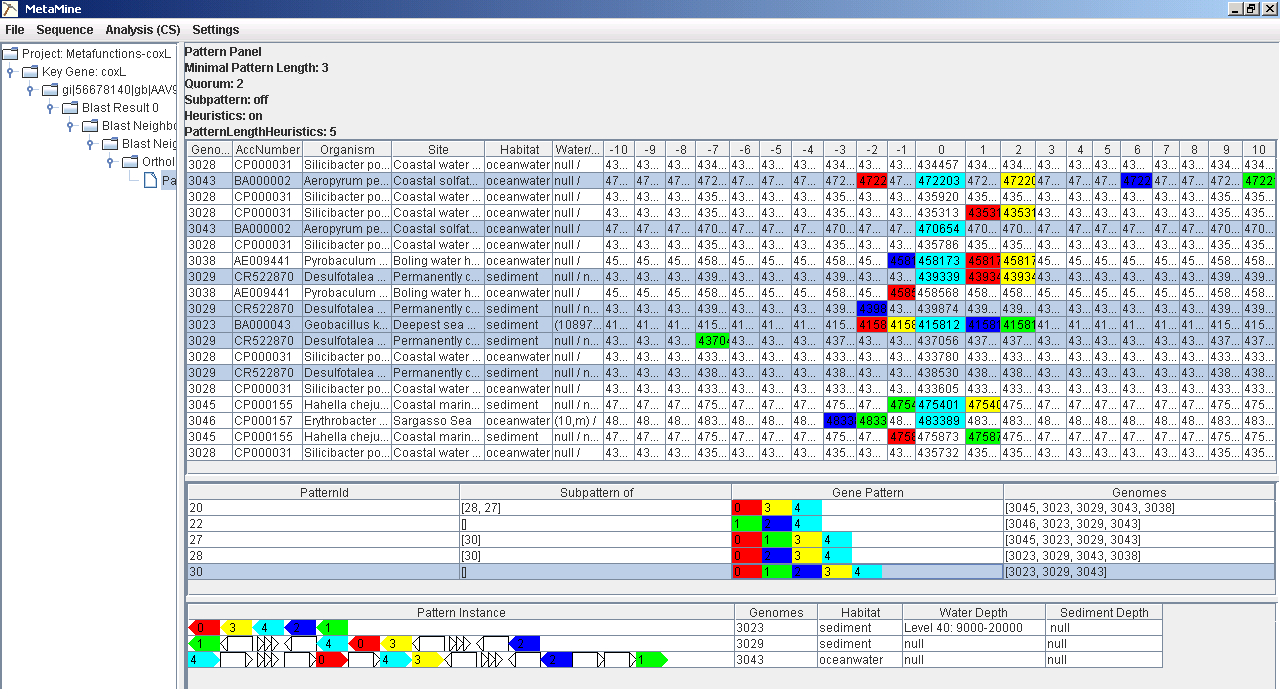


Next step: Save/Export project results

**9. Save/Export project results**

In order to save the project results all intermediate data can be exported to an XML file (see Fig 13). The stand-alone version of MetaMine can also save the project results into the local database.

Choose menu item “Export Project” from the File menu and specify a file name for your project.

Figure 13

**
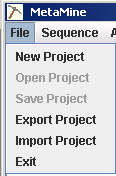

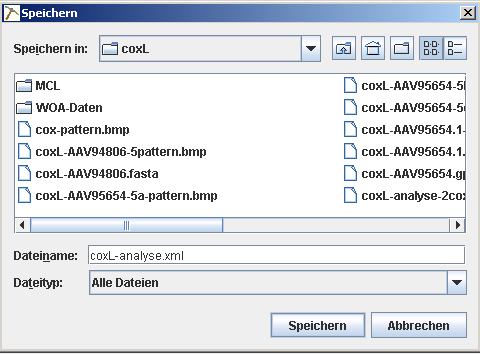
**

**Open/Import project results**

Choose menu item “Import Project” from the File menu in order to import your project data for further analysis. Select you file using the file open dialog.

**Reference:**

Tatusov RL, Galperin MY, Natale DA, Koonin EV: **The COG database: a tool for genome-scale analysis of protein functions and evolution**. *Nucleic Acids Research* 2000, **28**:33–36.

**Citation:**

Uta Bohnebeck, Thierry Lombardot, Renzo Kottmann and Frank Oliver Glöckner

MetaMine – A tool to detect and analyse gene patterns in their environmental context

Submitted to BMC Bioinformatics

MetaMine v1.2, released 09.07.2008
